# Habitat-related plastome evolution in the mycoheterotrophic *Neottia listeroides* complex (Orchidaceae, Neottieae)

**DOI:** 10.1186/s12870-023-04302-y

**Published:** 2023-05-27

**Authors:** Bing-Yi Shao, Mo-Zhu Wang, Si-Si Chen, Ji-Dong Ya, Xiao-Hua Jin

**Affiliations:** 1 State Key Laboratory of Systematic and Evolutionary Botany, Institute of Botany, Chinese Academy of Sciences, Beijing, China; 2 University of Chinese Academy of Sciences, Beijing, China; 3 Germplasm Bank of Wild Species, Kunming Institute of Botany, Chinese Academy of Sciences, Lanhei Road 132, Heilongtan, Kunming, 650201 Yunnan China

**Keywords:** Mycoheterotrophy, *Neottia listeroides* complex, Chloroplast genomes, Micro-evolution

## Abstract

**Background:**

Mycoheterotrophs, acquiring organic carbon and other nutrients from mycorrhizal fungi, have evolved repeatedly with substantial plastid genome (plastome) variations. To date, the fine-scale evolution of mycoheterotrophic plastomes at the intraspecific level is not well-characterized. A few studies have revealed unexpected plastome divergence among species complex members, possibly driven by various biotic/abiotic factors. To illustrate evolutionary mechanisms underlying such divergence, we analyzed plastome features and molecular evolution of 15 plastomes of *Neottia listeroides* complex from different forest habitats.

**Results:**

These 15 samples of *Neottia listeroides* complex split into three clades according to their habitats approximately 6 million years ago: Pine Clade, including ten samples from pine-broadleaf mixed forests, Fir Clade, including four samples from alpine fir forests and Fir-willow Clade with one sample. Compared with those of Pine Clade members, plastomes of Fir Clade members show smaller size and higher substitution rates. Plastome size, substitution rates, loss and retention of plastid-encoded genes are clade-specific. We propose to recognized six species in *N. listeroides* complex and slightly modify the path of plastome degradation.

**Conclusions:**

Our results provide insight into the evolutionary dynamics and discrepancy of closely related mycoheterotrophic orchid lineages at a high phylogenetic resolution.

**Supplementary Information:**

The online version contains supplementary material available at 10.1186/s12870-023-04302-y.

## Introduction

Mycoheterotrophs possess the ability to survive by acquiring nutrients from mycorrhizal fungi, relaxing the dependence on their own photosynthesis for carbon fixation [1–6]. Mycoheterotrophs commonly undergo loss or pseudogenization of photosynthesis-related genes due to relaxed selective constraints, further leading to dramatic plastid genome (plastome) reductions and structural rearrangements [1, 4, 7–14]. Moreover, there are repeated plastome-based phylogenetic changes during trophic transitions from autotrophy, via partial mycoheterotrophy (mixotrophy), to holomycotrophy [15, 16].

The loss of plastid-encoded genes coinciding with or following the evolution of parasitic plants has been extensively explored [1, 12, 16–19]. Recently, an increasing number of studies revealed more and more evolution details by incorporating dense sampling of plastomes across higher taxonomic levels (e.g. genera, families or tribes) containing parasitic plants, from the phylogenetic-comparative perspective [8, 15, 16, 19–25]. Plastomes of parasitic plants are characterized by elevated substitution rates, gene pseudogenization and loss. The path of plastome degradation in parasitic plants, proposed and revised based on syntheses of previous plastome evolution in parasitic plants, includes the following major stages: (1) loss and/or pseudogenization in the *ndh* genes complex; (2) loss and/or pseudogenization of photosynthesis genes; (3) loss and/or pseudogenization of photosynthesis-related genes with secondary functions, including *atp* genes; (4) loss and/or pseudogenization of other genes, such as *accD, ycf1, ycf2*; and (5) nearly complete or complete loss of the plastome [3, 19, 26, 27]. This model of plastome evolution has been observed in subsequent studies (such as [14, 19, 28–32]). Nevertheless, these studies may leave phylogenetic sampling or evolutionary route ‘gaps’ due to large temporal- and spatial-scale patterns [20]. By now, comparative plastome analyses at a fine scale, such as infrageneric or intraspecific levels, are rather scant [20]. These limited studies have revealed unexpectedly high plastome divergence among mycoheterotrophic species complex members, which is presumably related to mycorrhizal interactions, geographical barriers, or other biotic/abiotic factors [20]. Adaptive processes underlying such divergence, however, remain largely unknown [20]. Therefore, it is necessary to explore plastome variation at higher phylogenetic resolution and across diverse habitats to exquisitely illustrate plastome evolution of mycoheterotrophs.

**Fig. 1:**
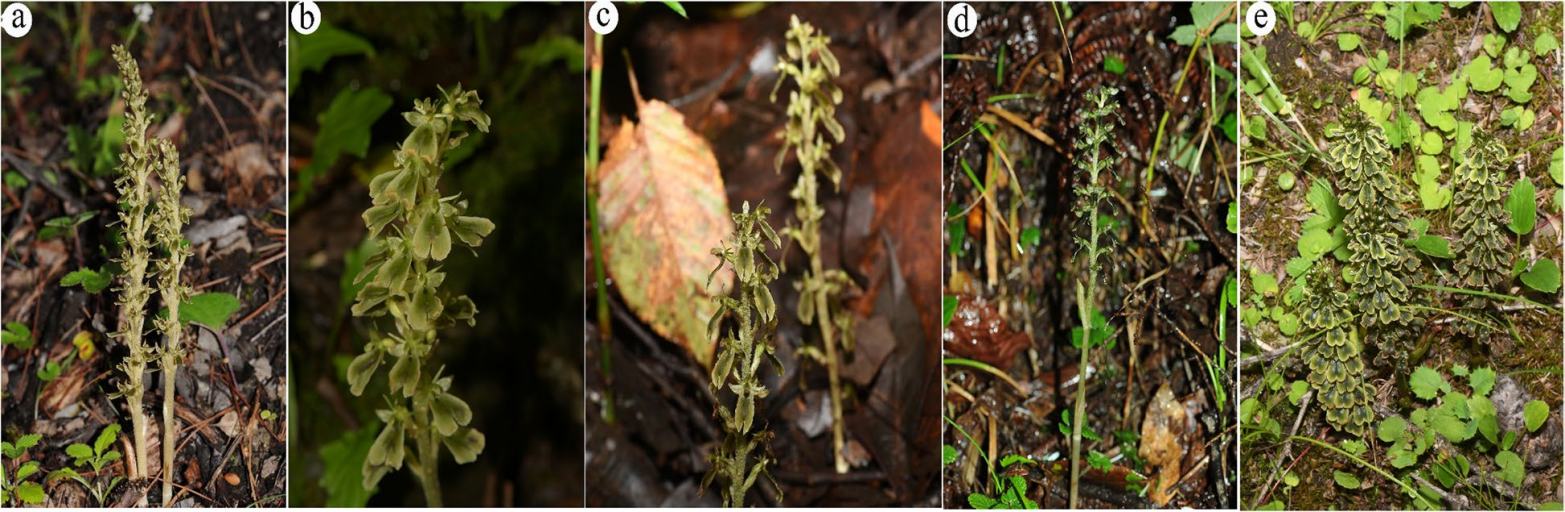
Members of the *Neottia listeroides* complex collected from different sites. **a** *N. listeroides* (LCY), **b** *N. megalochila* (LGZ), **c** *N. naungmungensis* (MMD), **d** *N. listeroides* (LGS), and **e** *N. megalochila* (MLJ)

*Neottia* Guettard (Orchidaceae, Neottieae) comprises approximately 73 species, including both autotrophic and mycoheterotrophic species and are widely distributed in northern temperate regions and alpine areas of Asian subtropical regions [2, 15, 18, 31, 33–42] (https://powo.science.kew.org/results?q=Neottia). Previous studies indicated that leafless *Neottia* are fully mycoheterotrophic and have evolved from leafy species only once in *Neottia* [15, 32, 43]. *N. listeroides* complex is composed of approximately six morphologically similar leafless holomycotrophic species (Fig. 1), including *N. listeroides*, *N. megalochila*, *N. Microglottis*, *N. smithianus* and *N. tenii* [44, 45]. These species generally occur in forests dominated by *Abies* or *Pinus* in temperate and alpine region of subtropical area of China (Yunnan, Sichuan, Shaanxi, and Tibet), Myanmar, Nepal, and neighboring regions (Table 1, Table S1) [44, 45]. In this study, we analyzed plastomes of *N. listeroides* complex members using densely sampling from different habitats, to explore their variation patterns and effects of environmental factors on micro-evolution of closely related mycoheterotrophic taxa.

**Table 1:** Sampling information of *Neottia listeroides* complex

Sample ID	Species	Location	Latitude (N)	Longitude (E)	Elevation (m)	Habitat	Voucher (Herbarium)
HHL01	*Neottia smithianus*	Yan'an, Shaanxi, China	35° 39′ 09"	108° 40′ 07"	1204	Pine-broadleaf mixed forest	19cs18561 (KUN)
HHL02	*Neottia smithianus*	Yan'an, Shaanxi, China	35° 39′ 34"	108° 31′ 18"	1540	Pine-broadleaf mixed forest	19cs18562 (KUN)
MMD	*Neottia naungmungensis*	Namaut, Chin, Myanmar	21° 12′ 44"	94° 00′ 15"	2344	Pine-broadleaf mixed forest	Jin X.H. 24,630 (PE)
LJL01	*Neottia listeroides*	Shigatse, Tibet, China	28° 21′ 57"	85° 19′ 55"	2714	Pine-broadleaf mixed forest	18cs17622 (KUN)
LJL02	*Neottia listeroides*	Shigatse, Tibet, China	28° 21′ 58"	85° 19′ 54"	2723	Pine-broadleaf mixed forest	18cs17621 (KUN)
LCY01	*Neottia listeroides*	Nyingchi, Tibet, China	29° 01′ 49"	97° 22′ 27"	2908	Pine-broadleaf mixed forest	Jin et al. SETET355 (PE)
LLZ01	*Neottia listeroides*	Nyingchi, Tibet, China	29.7369392	94.105065	3300	Pine-broadleaf mixed forest	Jin et al. ST2246 (PE)
LLZ02	*Neottia listeroides*	Nyingchi, Tibet, China	29° 46′ 52.28"	93°51′33.65"	3169	Pine-broadleaf mixed forest	Jin X.H. 38,341 (PE)
HLZ	*Neottia smithianus*	Nyingchi, Tibet, China	29°8′54.28"	94°12′25.12"	2981	Pine-broadleaf mixed forest	Jin X.H. 38,351 (PE)
MLJ	*Neottia megalochila*	Lijiang, Yunnan, China	27° 10′ 10"	100° 15′ 16"	3240	Alpine fir forest	Han Z.D. HZD002 (KUN)
LLJ	*Neottia megalochila*	Lijiang, Yunnan, China	27° 10′ 28"	100° 13′ 44"	3600	Alpine fir forest	Jin X.H. 23,453 (PE)
LGS	*Neottia listeroides*	Nujiang, Yunnan, China	27.9872034	98.5532579	3300	Alpine fir forest	Jin X.H. ST2246 (PE)
LGZ	*Neottia megalochila*	Garzê, Sichuan, China	29° 27′ 20"	101° 52′ 38"	3400	Alpine fir forest	Jin X. H. 20,298(PE)
HWQ	*Neottia listeroides*	Leiwuqi, Tibet, China	31°8′14.37"	96°33′45.1"	4118	Alpine fir forest–broadleaf mixed forest	Jin X. H. 38,248 (PE)
LCY02	*Neottia listeroides*	Chayu, Tibet, China	29°2′ 48.2"	97°20′35.6"	2900–3000	Pine-broadleaf mixed forest	Jin X. H. 31,524 (PE)

## Results

### Phylogenetic relationships and molecular dating of *Neottia listeroides* complex

**Fig. 2:**
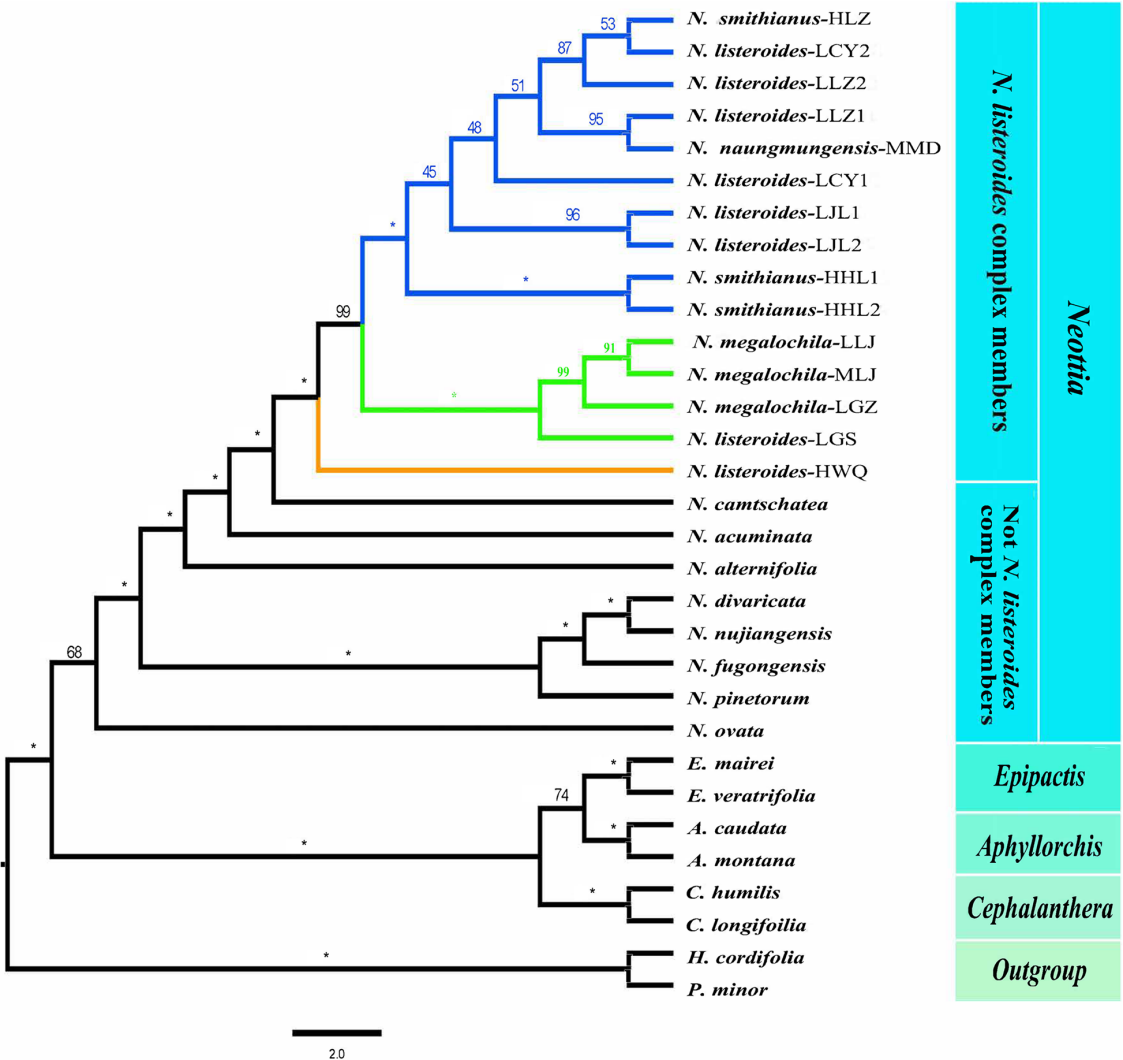
Phylogenetic relationships based on maximum likelihood (ML) analysis of mitochondrial protein-coding sequences (mtCDS). Numbers above branches represent bootstrap support (* indicates 100%). *N. listeroides* complex members were divided into three clades: Pine Clade (indicated by blue) including samples from pine-broadleaf mixed forests; Fir Clade (indicated by the green) including samples from alpine fir forests; and Fir-willow Clade (indicated by the yellow)

**Fig. 3:**
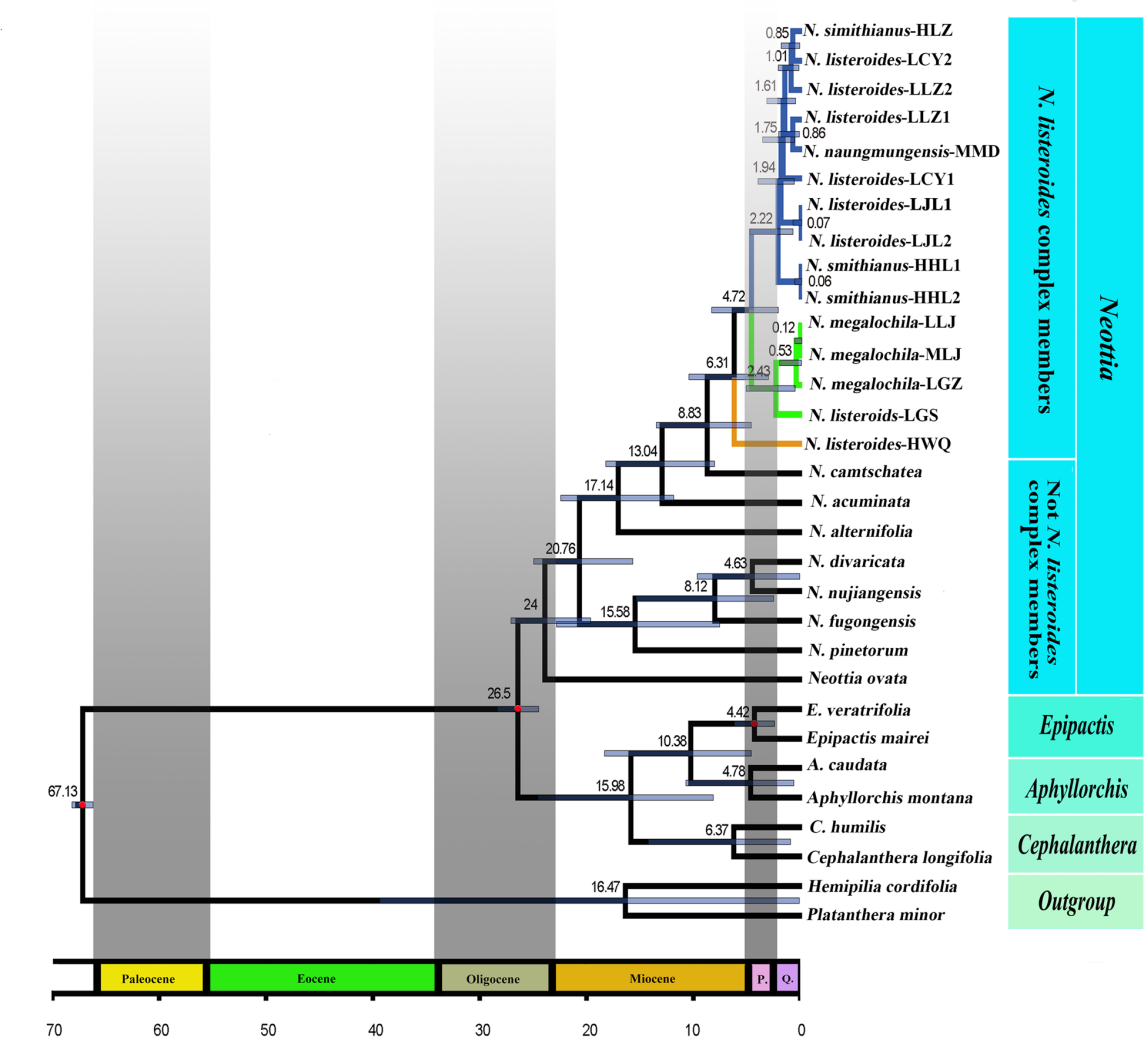
Time-calibrated phylogram based on concatenated sequences of all mtCDS. Numbers at nodes are median ages in million years ago (Ma). Pine Clade indicated by blue; Fir Clade indicated by the green; and Fir-willow Clade indicated by the yellow. The schematic diagrams of record of Earth’s climate was edited according to reported references [107]

Topologies from the phylogenetic analyses of protein coding genes of mitochondrial genomes (Fig. 2) and plastome genomes (Fig. S1) are different about the phylogenetic position and the interrelationships of *Neottia*. We here use the phylogram from mitochondrial genomes for subsequent analyses and discussion. The *N. listeroides* complex, along with other mycoheterotrophic species, such as *N. acuminata*, *N. camtschatea*, and *N. alternifolia*, formed a monophyletic group nesting within *Neottia* and sister to the clade consisting of *N. divaricata*, *N. nujiangensis*, *N. fugongensis*, *N. pinetorum*, and *N. ovata* (Fig. 2). Mycoheterotrophic species of *Neottia* evolved from the autotrophic plants approximately 13.04 million years ago (Ma) (Fig. 3). The *N. listeroides* complex evolved approximately 8.83 Ma and was subdivided into three clades according to forest habitats: Clade I, including ten samples (i.e. HHL-1, HHL-2, HLZ, *N. smithianus*; LJL1, LJL2, LCY1, LCY2, LLZ1, and LLZ2, *N. listeroides*; MMD, *N. naungmungensis*) from pine-broadleaf mixed forests; Clade II, including four samples (i.e. MLJ, LGZ, LLJ, *N. megalochila*; LGS, *N. listeroides*) from alpine fir forests (Fig. 3), and Clade III, including one sample (HWQ, *N. listeroides*) from alpine fir-willow forests. We thus designated the three phylogenetic clusters, Clade I, Clade II and Clade III as Pine Clade, Fir Clade, and Fir-willow Clade, respectively. Fir-willow Clade diverged from other two clades approximately 6.31 Ma, Pine Clade diverged from Fir Clade approximately 4.72 Ma (Fig. 3).

### General plastome features

Plastome size of 15 members of the *N. listeroides* complex range from 94,499 bp to 110,855 bp, with guanosine-cytosine (GC) content varies from 37.2% to 37.8%. All these plastomes possess the typical quadripartite structure, consisting of a pair of IR regions (26,145–27,814 bp) separated by LSC (35,272–48,291 bp) and SSC (6,500–9,932 bp). The number of annotated genes is 80 to 84, including 34–38 coding sequences (CDS), eight rRNA genes, and 38 tRNA genes (Table 2). In brief, within the *N. listeroides* complex, Fir-willow Clade has the largest plastome size, Fir Clade has the smallest plastome size. According to nucleotide diversity (Pi > 0.1), mutation hotspots among 15 samples are largely located in intergenic spacers, including *trnC-trnD*, *trnY-trnT*, *trnM-atpB*, *rbcL-accD*, and *rps15*-*ccsA* (Fig. S2). When the sample of *N. listeroides* (LCY1) was selected as the reference, sequences of other members from Pine Clade showed greater synteny to it, and divergence of three samples of *N. megalochila* (LLJ, LGZ, MLJ) one sample of *N. listeroides*(LGS) from the reference mainly occurred in intergenic non-coding regions with frequent deletions (Fig. S3).

**Table 2:** Chloroplast genome features of the *Neottia listeroides* complex

Sample ID	Species	Mean coverage	Length (bp)				GC Content (%)	Number of genes			
			Total	LSC	SSC	IR		Total	CDS	rRNA	tRNA
HHL01	*Neottia smithianus*	216.3	109,294	43,983	9935	27,688	37.3	82	36	8	38
HHL02	*Neottia smithianus*	272.3	108,902	43,570	9932	27,700	37.3	81	35	8	38
MMD	*Neottia naungmungensis*	230.6	106,552	42,071	9441	27,520	37.4	81	35	8	38
LJL01	*Neottia listeroides*	296.2	107,466	42,781	9441	27,622	37.3	80	34	8	38
LJL02	*Neottia listeroides*	379.2	107,466	42,781	9441	27,622	37.3	80	34	8	38
LCY01	*Neottia listeroides*	71.8	110,246	45,021	9597	27,814	37.2	81	35	8	38
LLZ01	*Neottia listeroides*	177.8	108,572	43,868	9702	27,501	37.3	81	35	8	38
MLJ	*Neottia megalochila*	266	98,947	39,543	6500	26,452	37.7	81	35	8	38
LGS	*Neottia listeroides*	227.5	94,499	35,272	6937	26,145	37.8	82	36	8	38
LGZ	*Neottia megalochila*	256.1	98,983	38,593	6514	26,438	37.6	84	38	8	38
LLJ	*Neottia megalochila*	336.9	98,975	39,597	6600	26,389	37.7	84	38	8	38
HLZ	*Neottia smithianus*	350	108,607	43,851	9594	27,581	37.3	81	35	8	38
HWQ	*Neottia listeroides*	376	110,855	48,291	8378	27,032	36.8	81	35	8	38
LLZ02	*Neottia listeroides*	420	107,116	42,448	9556	27,556	37.4	81	35	8	38
LCY02	*Neottia listeroides*	632	109,780	44,940	9624	27,608	37.2	81	35	8	38

*LSC* Large single copy, *SSC* Small single copy, *IR* Inverted repeats, *CDS* Protein-coding sequences

**Fig. 4:**
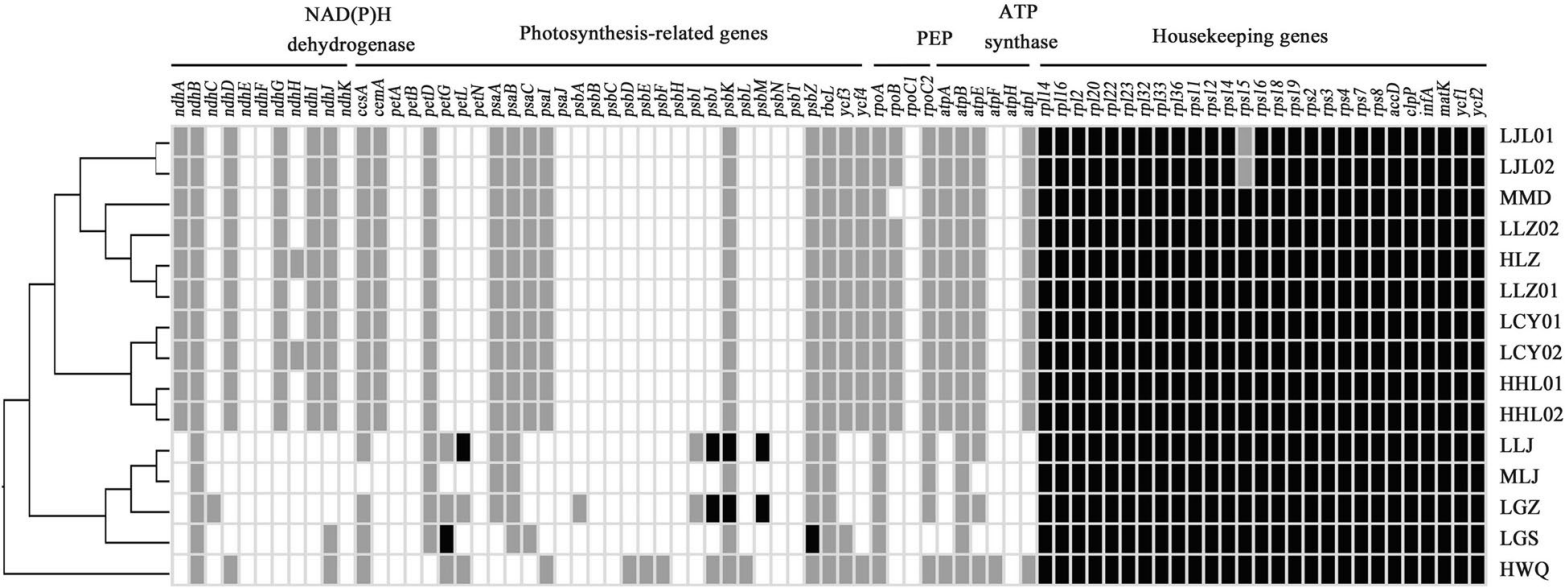
Protein-coding genes in *N. listeroides* complex. Black, grey and white boxes respectively represent intact genes, pseudogenes and gene loss. PEP = plastid-encoded RNA polymerase

A consistent gene order, without rearrangement, is maintained among all complex members (Fig. S4). At the junction of LSC/IRa (JLA), *trnK-UUU* flanked the JLA for Pine Clade samples, whereas it is located completely within the LSC for Fir Clade samples (Fig. S5). Genes encoding thylakoid NAD(P)H dehydrogenase (*ndh*), plastid-encoded RNA polymerase (PEP; *rpo*), and thylakoid ATP synthase (*atp*), as well as photosynthesis-related genes (*ccsA*, *cemA*, *pet*, *psa*, *psb*, *rbcL*, and *ycf3* and *ycf4*), are largely pseudogenized or deleted in all 15 samples, except retention of a few genes of photosystem II (*psbJ*, *psbK*, and *psbM* in LLJ and LGZ of *N. megalochila*, and *psbZ* in LGS of *N. listeroides* and cytochrome b6f complex (*petG* in LGS of *N. listeroides* and *petL* in LLJ of *N. megalochila*) in some Fir Clade samples (Fig. 4). Housekeeping genes have intact ORFs in almost all samples, although LJL-1 and LJL-2 (*N. listeroides*) functionally lost the ribosomal protein gene *rps15*. Overall, samples assigned to Pine Clade showed high similarity in gene content.

Members of the *N. listeroides* complex are split into three subgroups, i.e., Pine Clade, Fir Clade, and Fir-willow clade approximately 6.31 and 4.72 Ma, respectively. These three clades differ greatly in gene content, substitution rates and size of plastomes. Fir-willow and Pine clades have lost or pseudogenized all genes related to photosynthesis. Fir Clade retains a few photosynthesis-related genes, such as *pet* and *psb*, displaying substantial variations in gene content among individuals. RNA polymerase gene *rpoB* had been deleted from MMD (*N. naungmungensis*) and ribosomal protein gene *rps15* pseudogenized in LJL-1 and LJL-2 (*N. listeroides*) in Pine Clade. Fir Clade members show significantly reduced plastome size in comparison with two other clades.

### Molecular evolution

Fir-willow Clade has the lowest substitution rates in *N. listeroides* complex, whereas Fir Clade had highest evolutionary rates (Table S2, Fig. S6). Using *N. fugongensis* as a reference, all *N. listeroides* complex members are under purifying selection, with the signature of negative selection in most genes (ω < 0.5). However, selective constraint on some of genes are relaxed in Pine Clade (e.g. *rpl33*) and Fir Clade (e.g. *rpl22*, *rpl32*, *rps8*, and *clpP*) (Table S3). The branch-site model detected positively selected sites (BEB probability > 0.95) in five genes (i.e. *accD*, *matK*, *rpl20*, *rps11*, and *ycf2*) when Pine Clade was set as the foreground branch, while in two genes (i.e. *accD* and *rpl32*) as Fir Clade was the foreground branch (Table S4). Biased codon usage (RSCU > 1) existed in most amino acids, except tryptophan (Trp) and methionine (Met). Significance of difference in RSCU values among three clades was estimated using the *t*-test. Eight of 32 frequently used codons were underused in Fir Clade and Fir-pillow Clade sample compared with Pine Clade samples, whereas seven codons were overused (Table S5).

## Discussion

### Taxonomic treatment of *N. listeroides* complex

The taxonomy of fully mycoheterotrophic group, such as *Gastrodia* and *Neottia*, has been notoriously difficult due to greatly reduced plant body, few specimens that was usually in poor condition, variation of floral characters and the rarity of specimens [46]. The number of identified species of *Gastrodia* has been triple in last two decades with the help of digital cameras, molecular systematics and botanic survey [47–52]. The taxonomy of *N. listeroides* complex has been confused for a long time [44]. *N. smithianus* and *N. microglottis* were even transferred to another genus, *Holopogon* [53, 54]. *N. microglottis* and *N. tenii* have not been discovered in field since they were described. Phylogenomics indicated that all samples of *N. megalochia, N. naungmungensis* and *N. smithianus,* were nested within *N. listeroides.* Two samples of *N. smithianus* (LJL1, LJL2) were nested within Pine clade and diverged from other members of Pine Clade about 2.2 Ma. The third sample of *N. smithianus* diverged from *N. listeroides* less than 1 Ma. Three samples of *N. megalochila* belong to Fir clade and diverged from *N. listeroides* (LGS) within Fir Clade about 2.43 Ma. *N. naungmungensis* diverged from *N. listeroides* (LLZ1) less than 1 Ma and is characterized by its ecological niches and morphological characters (Table 1, Fig. 1). *N. listeroides* complex was rapid diversification in icehouse period of last 5 Ma, however, there is the discrepancy between the molecular systematic tree and the morphology. *N. listeroides(* HWQ) is morphological stasis although it diverged very early. Instead, some recent evolved sampling (such as MMD, LGZ, LLJ) differ greatly from *N. listeroides*. This discrepancy might have been contributed by many factors, such as incomplete lineage sorting, hybridization or molecular evolution rates.

Based on these, we tentatively propose to recognize approximately six species in this complex, i.e., *N. listeroides*, *N. megalochia*, *N. microglottis, N. naungmungensis* sp. nov. (MMD from Naungmung Mountains, Chin state, Myanmar), *N. smithianus* and *N. tenii*.* Neottia naungmungensis* differs from its relatives by its elliptic lip with about 2.5 cm long, apex bilobed, lobelets acute, and stigma lateral with stalk. *N. listeroides* is characterized by narrowly obovate-oblong lip about 3-4 mm wide, apex bilobed, lobelet apex acute or obtuse; *N. megalochila* by obovate lip about 6-10 mm wide, apex bilobed, lobelet apex truncate-rounded; *N. smithianus* by terminal stigma and anther more or less with filament; *N. tenii* differs from other species by its lip with a pair of auricles at base [44]. *N. microglottis* is characterized by its entire lip not bilobed at apex, however, the taxonomic identity remains to be confirmed. Species delimitation is urgently needed in this complex based on more sampling across the distribution range.

### Factors driving plastome diversification

Our results demonstrated that plastome structure and substitution rates of closely related mycoheterotrophic lineages within a species complex could diverge rapidly based on forest habitat types. Fully mycoheterotrophic orchids uptake organic carbon and essential nutrients from their mycorrhizal fungi that concurrently liaise with surrounding trees, thus forming tripartite symbiotic associations [55–57]. In this system, green trees are ultimate energy sources, and subsequently shape their microflora [55, 58]. As a consequence, diversity and composition of mycorrhizal symbionts at the local-scale vary substantially along with changes in forest dominant trees [56, 59, 60].

In this study, alpine fir forests are simply dominated by *Abies fabri*, whereas pine-broadleaf forests are characterized by more abundant ectomycorrhizal hosts, mainly composed of *Quercus* (e.g. *Q. mongolica*) and *Pinus* (e.g. *P. densata* and *P. yunnanensis*). Given that tree host diversity contributes to ectomycorrhizal fungi (ECM) richness, alpine fir forests are expected to possess fewer ECM fungal species than pine-broadleaf forests [61]. Together, we infer that, although the key ECM taxa colonizing *N. listeroides* complex members remain largely the same (possibly as ectomycorrhizal Sebacinales Clade A), the specificity of ECM associates in Fir Clade samples is potentially higher than that in Pine Clade samples, further leading to forest-type-dependent plastome divergence of complex members. As mycorrhizal symbioses are indispensable for growth and survival of mycoheterotrophic orchids, additional studies are needed to clarify tripartite vegetation-fungus-orchid associations.

From the forgoing considerations, identity and specificity of mycorrhizal associations differ considerably among orchid taxa, and profoundly affect the distribution, evolution, and diversification of mycoheterotrophic orchids [16, 62–64]. Two ecologically different clades of Sebacinales have been found to predominate in various putative mycorrhizal associates of *Neottia* [39]. In particular, Sebacinales Clade A including ectomycorrhizal fungi (ECM) mainly connects with trees and mycoheterotrophic orchids (e.g. *N. nidus-avis* and *N. camtschatea*), whereas taxa in non-ectomycorrhizal Sebacinales Clade B commonly form rhizoctonia symbionts with green orchids (e.g. *N. ovata* and *N. cordata*) [42, 56, 65, 66]. Based on limited empirical evidence, shifts of association from rhizoctonia to ECM symbionts, with increased specificity of fungal partners, are potential steps in the sequential evolution from autotrophy to holomycotrophy in *Neottia* [2, 39, 59]. Moreover, variation in mycorrhizal specificity within a certain orchid species complex may contribute to fine-scale phylogenetic diversification [67, 68]. Recently, Suetsugu et al. (2022) showed that the use of different symbiotic microbiont can contribute to the diversification of species in mycoheterotrophic plants [46], which suggests that a shift in symbiotic microbiont may have played a role in the ecological speciation of these plants.

The Fir Clade have highest substitution rates among three clades, likely associated with changes in codon preference resulting from mutational bias or selection, since seven of 32 frequently used codons were overused in Fir Clade samples. Moreover, selection on codon usage bias related to translation efficiency might reflect adaptation of individuals to their environments [30, 69, 70]. Selective constraints are relaxed in more genes for Fir Clade (e.g. *rpl22*, *rpl32*, *rps8*, and *clpP*) than Pine Clade (e.g. *rpl33*). We detected the signature of positive selection in five genes (i.e. *accD*, *matK*, *rpl20*, *rps11*, and *ycf2*) with Pine Clade as the foreground branch, while in two genes (i.e. *accD* and *rpl32*) with Fir Clade as the foreground branch. These genes may function importantly during adaptation of complex members to different habitats, contributing to the divergence of Pine Clade and Fir Clade [71].

### Plastome evolution in parasitic plants

It is worth noting that all members of *Neottia listeroides* have green plants despite the degeneration of the plastomes. Previous results indicated that some leafless orchids with green stems or purple stems, such as *Corallorhiza trifida* [72, 73], *Cymbidium macrorhizon* [74–76], and *Limodorum abortivum* [77, 78], can perform photosynthesis in stems or fruits even in the absence of leaves. However, plastomes of *Cymbidium macrorhizon*, *Corallorhiza trifida* and *Limodorum abortivum* [79] have nearly intact photosynthesis and photosynthesis-related genes, instead, these genes were nearly lost or pseudogenized in plastomes of *Neottia listeroides* complex. It seems that all members of *Neottia listeroides* complex have lost the ability of photosynthesis and fully depend on symbiotic microbionts for organic carbon.

Recent studies on extremely reduced plastomes (minimal plastomes) of several parasitic plant groups, including *Epipogium* (Orchidaceae) [1], *Epirixanthes* (Polygalaceae) [14], *Exacum* (Gentianceae) [28], Gastrodieae (Orchidaceae) [12] and *Sciaphilla* (Triuridaceae) [80, 81], reveal little known evolutionary trends, including the formation of *rrn* gene block, the retention or even increase of gene copies, such as *accD*, *clpP*, *ycf 1*, and *ycf2*. Comparative genomics of nuclear genome of mycoheterotrophic *Gastrodia menghaiensis* and autotrophic orchids (*Apostasia zhenzhenica, Dendrobium officinale*, *Phalaenopsis equestris*) showed that genes involved synthesis and degradation of chlorophyll were absent in genome of *Gastrodia menghaiensis* [82]. However, nuclear encoded genes related to plastid biosynthesis of fatty acids, and hormones are intact or even increased in copies [82]. These suggest plastids play important role even in fully mycoheterotrophic species and the loss and/or pseudogenization of all “housekeeping” genes is very rare case. These minimal plastomes don’t belong to any stage proposed in previous studies [3, 19, 26, 27]. The plastome degradation in parasitic plants also display a highly lineage-specific manner in gene retentions, pseudogenization and loss even within genus or species [15, 16, 20, 73].

Therefore, we propose to slightly modify the path of plastome degradation in previous studies [3, 19, 26, 27, 81, 83–85]: (1) loss and/or pseudogenization in the *ndh* genes complex; (2) retention, pseudogenization and/or loss of photosynthesis and photosynthesis-related genes, including *atp* genes; (3) loss and/or pseudogenization genetic apparatus and Maturase K gene, such as *rpo, mat*K; (4) retention even expansion of gene copies of other genes, including *accD, rrn, ycf1* and *ycf 2* genes; and (5) nearly complete or complete loss of the plastid genome. Plastomes of *Cymbidium macrorhizon* [75] fall within stage 1, plastomes of *Corallorhiza trifida* [27] in stage 2, *Neottia listeroides* complex and *Rhizanthella gardneri* [7] in stage 3, instead, most minimal plastomes of *Epipogium* [1], *Thismia* [10, 86] and Gastrodieae [12] are in stage 4.

## Conclusions

We analyzed plastome evolution of the *N. listeroides* complex composed of 15 samples from different habitats via phylogenetic and comparative approaches, to explore fine-scale evolutionary dynamics and discrepancies of closely related mycoheterotrophic lineages. We detected the rapid diversification of plastomes in terms of structure, gene content, and evolutionary rates during the last 4 Ma. Unexpectedly, the observed divergence is closely related to forest habitats. We hypothesized that specificity of mycorrhizal fungal partners contributes to such divergence, which needs to be further demonstrated by empirical evidences. In addition to gene loss, plastome evolution involves intricate but coordinated nucleus-plastid interactions, such as transfer of genes to the nucleus via multiple steps [7, 26, 87, 88]. Such transfer processes are species- or lineage-specific [1]. Therefore, it is also necessary to deeply explore the nuclear genome variation in complex members to understand their diversification mechanisms.

## Materials and Methods

### Sampling, DNA extraction, and sequencing

Fifteen samples of *N. listeroides* complex were collected from thirteen sites covered with different vegetation (Table 1, Fig. 1). Total genomic DNAs from silica gel-dried materials were extracted using a modified CTAB method [89]. DNAs with a concentration higher than 100 ng/μl were sonicated into ~ 500 bp fragments (Covaris M220; Woburn, MA, USA). Libraries were prepared following the user’s manual of the NEBNext Ultra DNA Library Prep Kit (New England Biolabs, Ipswich, MA, USA). Paired-end sequencing was performed on the Illumina HiSeq 2500 platform (Illumina, Inc., San Diego, CA, USA).

### Assembly and annotation

For quality control (QC), raw data were processed using the NGS QC Toolkit v.2.3.3 [90] to trim adaptors and filter low-quality reads (PHRED < 20, length < 70 bp). Clean reads were mapped to the plastome of *Calanthe triplicata* (NC_024544.1) using Geneious v.10.1.2 (Biomatters, Inc., Auckland, New Zealand) with medium–low sensitivity in five iterations. Plastid contigs from consensus sequences were de novo assembled in VELVET [91] over a range of k-mer values from 37 to 45 with auto-adjustment for coverage cutoffs. Subsequently, contigs were combined into scaffolds. After inverted repeat (IR) boundaries identification via BLAST [92], all reads were mapped with high stringency to the draft chloroplast genome for assembly errors correction [15]. The assembled chloroplast genomes were annotated using Geneious with 70% identity to the *C. triplicate* reference sequence, and then were checked and corrected manually [15]. Non-triplet frame shifts and premature stop codons were considered as pseudogenes [15].

### Phylogenetic analyses and molecular dating

In addition to 15 members of the *N. listeroides* complex, we also included another 16 species (Table S1) to complement the phylogenetic analysis. Two species of Orchidoideae, *Hemipilia cordifolia* and *Platanthera minor*, were used as outgroup. To minimize effects of accelerated substitution rates in mycoheterotrophic plastomes on phylogenetic inference [4, 93], we used mitochondrial protein-coding sequences (mtCDS) for phylogenetic reconstruction [4, 93]. Clean reads were mapped to mitochondrial genomes of *Gastrodia elata* (MF070084-MF070102) and *Phoenix dactylifera* (NC_016740) to obtain mtCDS sequences. Thirty-eight mtCDS were assembled for each sampling (supplementary Table S6). All mtCDS extracted by Geneious were aligned in MAFFT with default settings [94], and were manually adjusted via Bioedit v.5.0.9 [95]. After examination of saturation, aligned sequences were concatenated into a single multi-gene supermatrix using PhyloSuite [96]. The phylogenetic tree was constructed based on Maximum Likelihood (ML) with 1000 standard bootstrap (BS) pseudoreplicates using IQtree v.2.1.2 [97]. The tree was visualized in FigTree v.1.4.3 [98]. The phylogenetic tree based on 26 common house-keeping genes (Table S3) of plastomes was constructed as above.

All mtCDS were selected for molecular dating. The ML tree calibrated via BEAST v.2.4.8 [99] was used as a topological constraint, with *Corybas taliensis*, *Cheirostylis yunnanensis*, and *Spiranthes sinensis* as outgroup. The lognormal relaxed clock was selected in MrModelTest v.2.3 [100]. One orchid fossil was set as a calibration point for crown clades: subtribe Goodyerinae, 15 Ma (mean: 0, sigma: 1.5). Priors were placed on the stem node of Neottieae (offset: 67.17 Ma, mean: 0, sigma: 1.0), and the most recent common ancestor of Neottiaeae (offset: 26.19 Ma, mean: 0, sigma: 1.0) based on previous results [93, 101]. We conducted two independent runs of Markov chain Monte Carlo (MCMC) searches, sampling every 10,000 generations over 20 million generations, with four non-independent chains used for each run. Log files were monitored in Tracer v.1.6 [102]. Convergence was determined according to distribution and effective sample size (ESS) > 200. A maximum clade credibility (MCC) chronogram was generated in TreeAnnotator v.1.7.5 [103] with median heights for node ages. The time tree was visualized using FigTree.

### Plastome structure

Boundaries between single-copy regions (LSC and SSC) and inverted repeats (IR) regions (i.e. IR/SSC and IR/LSC) of each sample were visualized in Geneious. All 15 plastomes, excluding one IR in each sample, were compared by mVISTA (http://genome.lbl.gov/vista/mvista/submit.shtml) with the Shuffle-LAGAN model; the annotation for LCY (KU551272) was used as a reference. Nucleotide diversity (Pi) was calculated to identify mutation hotspots of the *N. listeroides* complex by a sliding window analysis via DnaSP v.6.10.04 [104]. The step size was set as 200 bp, with a 500 bp window length. Collinearity for 11 chloroplast genomes was evaluated through progressiveMauve algorithm in Geneious to identify syntenic blocks and visualize structural rearrangements.

### Detection of selection

To estimate non-uniform synonymous codon usage in protein-coding genes of complex members, the relative synonymous codon usage (RSCU) was calculated using CodonW v.1.4.2 (http://codonw.sourceforge.net/). RSCU < 1 denotes lack of usage bias, whereas RSCU > 1 indicates that a codon is overrepresented. A total of 34 retained protein-coding genes from the 15 plastomes were involved in selective pressure analyses. Single-copy CDS sequences without stop codons were aligned at the codon level using MUSCLE (codon) in MEGA v.7.0.2 [105]. The tree topology was inferred as described above. Non-synonymous (*d*_N_) and synonymous (*d*_S_) substitution rates indicated by branch lengths were estimated using PAML v.4.8 [106] under the branch model (run model = 0, model = 1, NSsites = 0) in the CODEML module [15].

Pairwise comparisons between samples were conducted via the pairwise model (runmode = 2, NSsites = 0), with *Neottia fungongensis* as the reference, followed by calculation of the selection intensity parameter ω (i.e. *d*_*N*_/*d*_*S*_). The codon frequencies were determined by the F3 × 4 model [1]. Significance of the ω parameter was determined by likelihood ratio tests (LRT), with Bonferroni correction for multiple comparisons. Values of ω > 1, ω ≈ 1, and ω < 1 suggest positive, neutral, and negative (purifying) selection, respectively (Yang & Nielsen, 2002).

Our phylogenetic analysis revealed that *N. listeroides* complex members could be separated into three clades designated as Pine, Fir and Fir-willow Clade (see RESULTS). To identify positively selected genes potentially functioning in adaptation of each group, the branch-site model was performed (model = 3, NSites = 3, fixed omega = 0, omega = 2) based on 31 protein-coding genes (excluding *rpl36*, *rps12*, and *rps7*). Pine Clade, Fir Clade and Fir-pillow Clade were respectively set as the foreground branch. Differences between the models and the null model M0 (model = 0, NSites = 0, assuming no site-wise or branch-wise *d*_N_/*d*_S_ variation) were evaluated by the LRT with a χ^2^ distribution at a threshold of *p* < 0.05. In addition, the Bayesian Empirical Bayes (BEB) method was used to identify specific sites under positive selection by calculating posterior probabilities. A gene with a high posterior probability (BEB > 0.95) and a *p*-value < 0.05 was considered as positively selected.

## Supplementary Information

**Additional file 1: Table S1.** Taxon sampling and GenBank accession numbers. **Table S2.** Synonymous (*d*_S_) and non-synonymous (*d*_N_) substitution rates of *N. listeroides *complex members, relative to the reference *N.fugongensis*. **Table S3.** Selection pressure on 26 “housekeeping genes” of each sample relative to the reference *N.fugongensis*. **Table S4.** Positively selected sites detected by the branch-sitesmodel. **Table S5.** Relative synonymous codon usage of* N. listeroides* complex plastomes. **Table S6.** Mitochondrial protein-coding genes in *N.listeroides* complex. **Fig. S1.** Phylogenetic relationships based on maximum likelihood (ML) analysis of protein-coding sequences of plastid genome (ptCDS). Numbers above branches represent bootstrap support. *N. listeroides *complex members were divided into three clades: Pine Clade (blue branch) including samples from pine-broadleaf mixed forests; Fir Clade (green branch) including samples from alpine fir forests and For Clade (yellow branch) including sample from alpine fir--broadleaf mixed forest. **Fig. S2.** Mutation hotspots in plastomes of the *N. listreoides* complex. **Fig. S3.** Sequence identity of plastomes of *N. listeroides* complex members (LCY-1 as the reference). The vertical scale represents the percentage of identity between 50% and 100%. The horizonal axis indicates the coordinates within the plastomes. **Fig. S4.** Colinear analysis of plastomes of the *N. listeroides* complex. Color bands are locally-collinear blocks, representing homologous gene clusters. Within each block, similarity profiles of sequences corresponding to the average conservative level was shown. **Fig. S5.** Comparison of LSC, SSC, and IR border regions among 15 *N. listeroides* complex plastomes. Colored boxes for genes represent the gene position. Gene and region lengths are not to scale. **Fig. S6.** Branch length of non-synonymous (dN) and synonymous (dS) substitution rates of *N. listeroides* complex members.

## Data Availability

All 15 newly sequenced and annotated plastid genomes generated in this study have been submitted to GenBase (https://ngdc.cncb.ac.cn/genbase) with accession number from C_AA002280.1 to C_AA002294.1. All sequenced data are available from GenBase (https://ngdc.cncb.ac.cn/genbase). The online resources of plastome data (NC_030712.1, NC_030711.1, NC_030710.1, KU551266.1, NC_030709.1, NC_030708.1, NC_030705.1, KU551263.1, NC_030706.1, NC_030703.1, MN416689.1) were downloaded from NCBI (https://www.ncbi.nlm.nih.gov/).
