# Supplementary material for: Habitat-related plastome evolution in the mycoheterotrophic Neottia listeroides complex (Orchidaceae, Neottieae)
Source: BMC Plant Biol. 2023 May 27;23:282. doi: 10.1186/s12870-023-04302-y (PMC10224299; doi:10.1186/s12870-023-04302-y)
Supplement: Supplementary file 1 — Additional file 1: Table S1. Taxon sampling and GenBank accession numbers. Table S2. Synonymous (dS) and non-synonymous (dN) substitution rates of N. listeroides complex members, relative to the reference N.fugongensis. Table S3. Selection pressure on 26 “housekeeping genes” of each sample relative to the reference N.fugongensis. Table S4. Positively selected sites detected by the branch-sitesmodel. Table S5. Relative synonymous codon usage of N. listeroides complex plastomes. Table S6. Mitochondrial protein-coding genes in N.listeroides complex. Fig. S1. Phylogenetic relationships based on maximum likelihood (ML) analysis of protein-coding sequences of plastid genome (ptCDS). Numbers above branches represent bootstrap support. N. listeroides complex members were divided into three clades: Pine Clade (blue branch) including samples from pine-broadleaf mixed forests; Fir Clade (green branch) including samples from alpine fir forests and For Clade (yellow branch) including sample from alpine fir--broadleaf mixed forest. Fig. S2. Mutation hotspots in plastomes of the N. listreoides complex. Fig. S3. Sequence identity of plastomes of N. listeroides complex members (LCY-1 as the reference). The vertical scale represents the percentage of identity between 50% and 100%. The horizonal axis indicates the coordinates within the plastomes. Fig. S4. Colinear analysis of plastomes of the N. listeroides complex. Color bands are locally-collinear blocks, representing homologous gene clusters. Within each block, similarity profiles of sequences corresponding to the average conservative level was shown. Fig. S5. Comparison of LSC, SSC, and IR border regions among 15 N. listeroides complex plastomes. Colored boxes for genes represent the gene position. Gene and region lengths are not to scale. Fig. S6. Branch length of non-synonymous (dN) and synonymous (dS) substitution rates of N. listeroides complex members. [file 12870_2023_4302_MOESM1_ESM.docx]

**Habitat-related plastome evolution in the mycoheterotrophic *Neottia listeroides* complex (Orchidaceae, Neottieae)**

Bing-Yi Shao^1,3,#^, Mo-Zhu Wang^1,#^, Si-Si Chen^1^, Ji-Dong Ya^2^, Xiao-Hua Jin^1,*^

^1^State Key Laboratory of Systematic and Evolutionary Botany, Institute of Botany, Chinese Academy of Sciences, Beijing, China

^2^Germplasm Bank of Wild Species, Kunming Institute of Botany, Chinese Academy of Sciences, Lanhei Road 132, Heilongtan, Kunming, Yunnan 650201, China

^3^University of Chinese Academy of Sciences, Beijing, China

#### * Corresponding author, Email: [xiaohuajin@ibcas.ac.cn](mailto:xiaohuajin@ibcas.ac.cn)

#### #These authors contributed equally.

Supplementary Material

Table S1. Taxon sampling and GenBank accession numbers.

| Sample ID | Species | Location | Plastid genome  GenBank accession | Voucher |
| --- | --- | --- | --- | --- |
| LJL1 | *Neottia listeroides* | Shigatse, Tibet, China | C_AA002287.1 | Ya J.D. 18CS17622（KUN） |
| LJL2 | *Neottia listeroides* | Shigatse, Tibet, China | C_AA002293.1 | Ya J.D. 18CS17621（KUN） |
| LCY1 | *Neottia listeroides* | Nyingchi, Tibet, China | C_AA002280.1 | Jin et al. SETET355（PE） |
| LLZ1 | *Neottia listeroides* | Nyingchi, Tibet, China | C_AA002285.1 | Jin X.H. 13283（PE） |
| LLZ2 | *Neottia listeroides* | Nyingchi, Tibet, China | C_AA002286.1 | Jin X.H. 38341（PE） |
| LGS | *Neottia listeroides* | Gongshan, Yunnan, China | C_AA002283.1 | Jin et al. ST2246（PE） |
| LGZ | *Neottia megalochila* | Garzê, Sichuan, China | C_AA002282.1 | Jin X.H. 20298（PE） |
| HWQ | *Neottia listeroides* | Leiwuqi, Tibet, China | C_AA002292.1 | Jin X.H. 38248（PE） |
| LCY2 | *Neottia listeroides* | Chayu, Tibet, China | C_AA002281.1 | Jin X.H. 31524（PE） |
| MMD | *Neottia naungmungensis,* sp.nov. | Namaut, Chin, Myanmar | C_AA002289.1 | Jin X.H. 246302（PE） |
| LLJ | *Neottia megalochila* | Lijiang, Yunnan, China | C_AA002284.1 | Jin X.H. 23453（PE） |
| MLJ | *Neottia megalochila* | Lijiang, Yunnan, China | C_AA002288.1 | Han Z.D. HZD19002（KUN） |
| HHL1 | *Neottia smithianus* | Yan'an, Shaanxi, China | C_AA002290.1 | Ya J.D. 19CS18561（KUN） |
| HHL2 | *Neottia smithianus* | Yan'an, Shaanxi, China | C_AA002294.1 | Ya J.D. 19CS18562（KUN） |
| HLZ | *Neottia smithianus* | Nyingchi, Tibet, China | C_AA002291.1 | Jin X.H. 38351（PE） |

**Table S2** Synonymous (*d*_S_) and non-synonymous (*d*_N_) substitution rates of *N. listeroides* complex members, relative to the reference *N. fugongensis*

|  | ω | *d*_N_ | *d_S_* |
| --- | --- | --- | --- |
| LJL01 | 0.4148 | 0.0187 | 0.045 |
| LJL02 | 0.4148 | 0.0187 | 0.045 |
| MMD | 0.4198 | 0.0188 | 0.0447 |
| LLZ02 | 0.4152 | 0.0186 | 0.0448 |
| HLZ | 0.4164 | 0.0186 | 0.0446 |
| LLZ01 | 0.4172 | 0.0186 | 0.0445 |
| LCY01 | 0.4112 | 0.0184 | 0.0448 |
| LCY02 | 0.414 | 0.0185 | 0.0447 |
| HHL01 | 0.4094 | 0.0182 | 0.0444 |
| HHL02 | 0.4106 | 0.0182 | 0.0442 |
| LLJ | 0.4077 | 0.0187 | 0.0457 |
| MLJ | 0.4058 | 0.0187 | 0.046 |
| LGZ | 0.4096 | 0.0186 | 0.0455 |
| LGS | 0.4194 | 0.0198 | 0.0472 |
| HWQ | 0.4157 | 0.0184 | 0.0441 |

**Table S3** Selection pressure on 26 “housekeeping genes” of each sample relative to the reference *N. fugongensis*

|  | LJL01 | LJL02 | MMD | LLZ02 | HLZ | LLZ01 | LCY01 | LCY02 | HHL01 | HHL02 | LLJ | MLJ | LGZ | LGS | HWQ |
| --- | --- | --- | --- | --- | --- | --- | --- | --- | --- | --- | --- | --- | --- | --- | --- |
| *infA* | 0.0010 | 0.0010 | 0.0010 | 0.0010 | 0.0010 | 0.0010 | 0.3900 | 0.3900 | 0.0010 | 0.0010 | 0.0010 | 0.0010 | 0.0010 | 0.0996 | 0.0010 |
| *rpl2* | 0.1632 | 0.1632 | 0.1632 | 0.1632 | 0.1632 | 0.1632 | 0.2117 | 0.2117 | 0.2117 | 0.2117 | 0.0721 | 0.0721 | 0.0721 | 0.0720 | 0.1512 |
| *rpl14* | 0.0848 | 0.0848 | 0.0848 | 0.0848 | 0.0848 | 0.0848 | 0.0848 | 0.0848 | 0.0848 | 0.0848 | 0.0819 | 0.0819 | 0.0819 | 0.0543 | 0.0883 |
| *rpl16* | 0.1643 | 0.1643 | 0.1643 | 0.1643 | 0.1643 | 0.1643 | 0.1643 | 0.1643 | 0.1643 | 0.1643 | 0.1643 | 0.1643 | 0.1643 | 0.2457 | 0.1781 |
| *rpl20* | 0.3540 | 0.3540 | 0.3540 | 0.3540 | 0.3540 | 0.3540 | 0.3017 | 0.3017 | 0.3017 | 0.3017 | 0.4181 | 0.4181 | 0.4181 | 0.4698 | 0.2856 |
| *rpl22* | 0.3322 | 0.3322 | 0.3322 | 0.3322 | 0.3322 | 0.3322 | 0.3322 | 0.3322 | 0.3322 | 0.3322 | 0.6877 | 0.6877 | 0.6877 | 0.6877 | 0.2615 |
| *rpl23* | 0.4114 | 0.4114 | 0.4114 | 0.4114 | 0.4114 | 0.4114 | 99.0000 | 99.0000 | 99.0000 | 99.0000 | 99.0000 | 99.0000 | 99.0000 | 99.0000 | 99.0000 |
| *rpl32* | 99.0000 | 99.0000 | 99.0000 | 99.0000 | 99.0000 | 99.0000 | 99.0000 | 99.0000 | 99.0000 | 99.0000 | 99.0000 | 99.0000 | 99.0000 | 1.2682 | 1.7704 |
| *rpl33* | 1.0434 | 1.0434 | 1.0434 | 1.0434 | 1.0434 | 1.0434 | 0.6167 | 0.6167 | 0.6167 | 0.6167 | 0.2068 | 0.2068 | 0.2068 | 0.2068 | 0.2254 |
| *rpl36* | 0.0010 | 0.0010 | 0.0010 | 0.0010 | 0.0010 | 0.0010 | 0.0010 | 0.0010 | 0.0010 | 0.0010 | 0.0010 | 0.0010 | 0.0010 | 0.0010 | 0.0010 |
| *rps2* | 0.1793 | 0.1793 | 0.1793 | 0.1793 | 0.1793 | 0.1793 | 0.1793 | 0.1793 | 0.1793 | 0.1793 | 0.1544 | 0.1544 | 0.1544 | 0.1651 | 0.2415 |
| *rps3* | 0.1840 | 0.1840 | 0.1840 | 0.1840 | 0.1840 | 0.1840 | 0.1840 | 0.1840 | 0.1840 | 0.1840 | 0.1662 | 0.1662 | 0.1662 | 0.1984 | 0.2191 |
| *rps4* | 0.3603 | 0.3603 | 0.3603 | 0.3603 | 0.3603 | 0.3603 | 0.3986 | 0.3986 | 0.3986 | 0.3986 | 0.3703 | 0.3703 | 0.3703 | 0.3918 | 0.5624 |
| *rps7* | 0.0010 | 0.0010 | 0.0010 | 0.0010 | 0.0010 | 0.0010 | 0.0010 | 0.0010 | 0.0010 | 0.0010 | 0.0010 | 0.0010 | 0.0010 | 0.0010 | 0.2693 |
| *rps8* | 0.3586 | 0.3586 | 0.3586 | 0.3586 | 0.3586 | 0.3586 | 0.3050 | 0.3050 | 0.3051 | 0.3051 | 0.9262 | 0.9262 | 0.9262 | 0.5667 | 0.6575 |
| *rps11* | 0.4268 | 0.4268 | 0.4806 | 0.4268 | 0.4268 | 0.4268 | 0.3603 | 0.3603 | 0.3586 | 0.3586 | 0.4328 | 0.4328 | 0.5903 | 0.5034 | 0.8834 |
| *rps12* | 0.0010 | 0.0010 | 0.0010 | 0.0010 | 0.0010 | 0.0010 | 0.0010 | 0.0010 | 0.0010 | 0.0010 | 0.0010 | 0.0010 | 0.0010 | 0.0010 | 0.0010 |
| *rps14* | 0.2089 | 0.2089 | 0.2089 | 0.2089 | 0.2089 | 0.2089 | 0.2089 | 0.2089 | 0.2089 | 0.2089 | 0.1590 | 0.1590 | 0.1590 | 0.2365 | 0.1867 |
| *rps16* | 0.1182 | 0.1182 | 0.1182 | 0.1182 | 0.1182 | 0.1182 | 0.1182 | 0.1182 | 0.1182 | 0.1182 | 0.1182 | 0.1182 | 0.1182 | 0.0957 | 0.0769 |
| *rps18* | 0.2574 | 0.2574 | 0.2574 | 0.2574 | 0.2666 | 0.2666 | 0.2574 | 0.3421 | 0.3060 | 0.3060 | 0.3122 | 0.3122 | 0.3122 | 0.2438 | 0.1820 |
| *rps19* | 99.0000 | 99.0000 | 99.0000 | 99.0000 | 99.0000 | 99.0000 | 99.0000 | 99.0000 | 99.0000 | 99.0000 | 0.8323 | 0.8323 | 0.8323 | 99.0000 | 99.0000 |
| *matK* | 0.2936 | 0.2936 | 0.3006 | 0.3098 | 0.2936 | 0.2936 | 0.2721 | 0.2867 | 0.2807 | 0.2807 | 0.2733 | 0.2733 | 0.2733 | 0.2379 | 0.3890 |
| *clpP* | 0.2547 | 0.2547 | 0.2547 | 0.2547 | 0.2547 | 0.2547 | 0.3162 | 0.3162 | 0.3162 | 0.3162 | 0.8303 | 0.8303 | 0.8303 | 0.6491 | 0.3236 |
| *accD* | 0.8141 | 0.8141 | 0.9812 | 0.9812 | 0.9812 | 0.9812 | 0.9358 | 0.9359 | 1.0050 | 1.0050 | 0.7863 | 0.7863 | 0.7863 | 0.7362 | 0.8587 |
| *ycf1* | 0.5159 | 0.5159 | 0.5204 | 0.5047 | 0.5190 | 0.5237 | 0.5214 | 0.5166 | 0.5214 | 0.5166 | 0.5371 | 0.5273 | 0.5371 | 0.4924 | 0.4895 |
| *ycf2* | 0.6044 | 0.6044 | 0.6117 | 0.6044 | 0.6044 | 0.6044 | 0.5617 | 0.5617 | 0.5540 | 0.5732 | 0.6227 | 0.6227 | 0.6227 | 0.7591 | 0.5590 |

**Table S4 Positively selected sites detected by the branch-sites model**

|  | Pine Clade as foreground | |  | Fir Clade as foreground | |
| --- | --- | --- | --- | --- | --- |
|  | *p*-value | BEB value |  | *p*-value | BEB value |
| *accD* | <0.005 | 121 T 0.976* 181 M 0.972* 182 E 0.997**  183 S 0.975* |  | <0.005 | 484 N 0.974* 485 Q 0.987* |
| *matK* | <0.05 | 66 S 0.951* 373 L 0.950* |  | / | / |
| *rpl20* | <0.005 | 126 K 0.996** 128 E 0.999** 129 Q 0.999** |  | / | / |
| *rpl32* | / | / |  | <0.005 | 48 L 0.995** 49 R 1.000** 50 Q 0.999** |
| *rps11* | <0.05 | 26 I 0.983* |  | / | / |
| *ycf2* | <0.005 | 1229 S 1.000** |  | / | / |

**Table S5 Relative synonymous codon usage of *N. listeroides* complex plastomes**

*Note*: RSCU values > 1 were highlighted in bold. Frequently used codons in red were overused in Fir Clade samples (i.e. MLJ, LGS, LGZ, and LLJ) when compared to Pine Clade samples (i.e. HHL-1, HHL-2, MMD, LJL-1, LJL-2, LCY, and LLZ), while in blue were underused.

|  |  | **LJL01** | **LJL02** | **MMD** | **LZZ01** | **HHL01** | **HHL02** | **LCY01** | **LLJ01** | **LLJ02** | **LGZ** | **LGS** | **HLZ** | **HWQ** | **LLZ02** | **LCY02** |
| --- | --- | --- | --- | --- | --- | --- | --- | --- | --- | --- | --- | --- | --- | --- | --- | --- |
| Ala | GCU | **1.528** | **1.498** | **1.504** | **1.509** | **1.509** | **1.488** | **1.498** | **1.491** | **1.514** | **1.519** | **1.519** | **1.5** | **1.52** | **1.5** | **1.49** |
|  | GCC | 0.542 | 0.551 | 0.539 | 0.541 | 0.541 | 0.561 | 0.537 | 0.545 | 0.556 | 0.571 | 0.571 | 0.6 | 0.59 | 0.6 | 0.63 |
|  | GCA | **1.486** | **1.498** | **1.504** | **1.495** | **1.495** | **1.502** | **1.512** | **1.505** | **1.472** | **1.463** | **1.463** | **1.44** | **1.47** | **1.45** | **1.43** |
|  | GCG | 0.444 | 0.452 | 0.454 | 0.456 | 0.456 | 0.449 | 0.452 | 0.459 | 0.458 | 0.446 | 0.446 | 0.45 | 0.43 | 0.45 | 0.45 |
| Arg | CGU | **1.186** | **1.184** | **1.184** | **1.183** | **1.183** | **1.186** | **1.18** | **1.165** | **1.151** | **1.163** | **1.163** | **1.15** | **1.15** | **1.16** | **1.16** |
|  | CGC | 0.355 | 0.357 | 0.357 | 0.363 | 0.363 | 0.361 | 0.356 | 0.351 | 0.36 | 0.35 | 0.35 | 0.37 | 0.36 | 0.37 | 0.37 |
|  | CGA | **1.236** | **1.235** | **1.224** | **1.215** | **1.215** | **1.247** | **1.22** | **1.258** | **1.223** | **1.245** | **1.256** | **1.18** | **1.15** | **1.19** | **1.2** |
|  | CGG | 0.466 | 0.469 | 0.469 | 0.457 | 0.457 | 0.464 | 0.468 | 0.423 | 0.442 | 0.432 | 0.422 | 0.49 | 0.45 | 0.49 | 0.49 |
|  | AGA | **2.169** | **2.153** | **2.153** | **2.17** | **2.17** | **2.144** | **2.166** | **2.216** | **2.209** | **2.213** | **2.213** | **2.15** | **2.21** | **2.15** | **2.13** |
|  | AGG | 0.588 | 0.602 | 0.612 | 0.612 | 0.612 | 0.598 | 0.61 | 0.588 | 0.616 | 0.597 | 0.597 | 0.65 | 0.68 | 0.65 | 0.65 |
| Asn | AAU | **1.563** | **1.563** | **1.566** | **1.56** | **1.56** | **1.566** | **1.558** | **1.577** | **1.573** | **1.575** | **1.576** | **1.54** | **1.57** | **1.55** | **1.55** |
|  | AAC | 0.437 | 0.437 | 0.434 | 0.44 | 0.44 | 0.434 | 0.442 | 0.423 | 0.427 | 0.425 | 0.424 | 0.46 | 0.43 | 0.45 | 0.45 |
| Asp | GAU | **1.629** | **1.633** | **1.638** | **1.638** | **1.638** | **1.642** | **1.641** | **1.642** | **1.641** | **1.638** | **1.638** | **1.65** | **1.66** | **1.65** | **1.65** |
|  | GAC | 0.371 | 0.367 | 0.362 | 0.362 | 0.362 | 0.358 | 0.359 | 0.358 | 0.359 | 0.362 | 0.362 | 0.35 | 0.34 | 0.35 | 0.35 |
| Cys | UGU | **1.492** | **1.486** | **1.491** | **1.486** | **1.486** | **1.491** | **1.491** | **1.477** | **1.464** | **1.468** | **1.468** | **1.47** | **1.49** | **1.47** | **1.47** |
|  | UGC | 0.508 | 0.514 | 0.509 | 0.514 | 0.514 | 0.509 | 0.509 | 0.523 | 0.536 | 0.532 | 0.532 | 0.53 | 0.51 | 0.53 | 0.53 |
| Gln | CAA | **1.58** | **1.579** | **1.579** | **1.575** | **1.575** | **1.576** | **1.583** | **1.559** | **1.548** | **1.557** | **1.557** | **1.53** | **1.51** | **1.53** | **1.53** |
|  | CAG | 0.42 | 0.421 | 0.421 | 0.425 | 0.425 | 0.424 | 0.417 | 0.441 | 0.452 | 0.443 | 0.443 | 0.47 | 0.49 | 0.47 | 0.47 |
| Glu | GAA | **1.486** | **1.487** | **1.483** | **1.483** | **1.483** | **1.489** | **1.485** | **1.5** | **1.499** | **1.497** | **1.497** | **1.44** | **1.45** | **1.43** | **1.44** |
|  | GAG | 0.514 | 0.513 | 0.517 | 0.517 | 0.517 | 0.511 | 0.515 | 0.5 | 0.501 | 0.503 | 0.503 | 0.56 | 0.55 | 0.57 | 0.56 |
| Gly | GGU | **1.144** | **1.143** | **1.147** | **1.143** | **1.143** | **1.14** | **1.141** | **1.166** | **1.167** | **1.204** | **1.204** | **1.12** | **1.19** | **1.12** | **1.12** |
|  | GGC | 0.425 | 0.429 | 0.416 | 0.418 | 0.418 | 0.427 | 0.414 | 0.399 | 0.399 | 0.408 | 0.408 | 0.38 | 0.36 | 0.38 | 0.38 |
|  | GGA | **1.722** | **1.724** | **1.716** | **1.714** | **1.714** | **1.72** | **1.717** | **1.719** | **1.726** | **1.682** | **1.682** | **1.74** | **1.75** | **1.73** | **1.74** |
|  | GGG | 0.709 | 0.704 | 0.721 | 0.724 | 0.724 | 0.712 | 0.727 | 0.716 | 0.708 | 0.706 | 0.706 | 0.77 | 0.7 | 0.78 | 0.76 |
| His | CAU | **1.584** | **1.582** | **1.578** | **1.584** | **1.584** | **1.582** | **1.578** | **1.588** | **1.612** | **1.59** | **1.59** | **1.57** | **1.58** | **1.56** | **1.57** |
|  | CAC | 0.416 | 0.418 | 0.422 | 0.416 | 0.416 | 0.418 | 0.422 | 0.412 | 0.388 | 0.41 | 0.41 | 0.43 | 0.42 | 0.44 | 0.43 |
| Ile | AUU | **1.391** | **1.397** | **1.39** | **1.383** | **1.383** | **1.418** | **1.394** | **1.398** | **1.391** | **1.403** | **1.403** | **1.37** | **1.39** | **1.37** | **1.39** |
|  | AUC | 0.583 | 0.589 | 0.594 | 0.597 | 0.597 | 0.586 | 0.596 | 0.584 | 0.601 | 0.583 | 0.583 | 0.64 | 0.61 | 0.64 | 0.63 |
|  | AUA | **1.026** | **1.014** | **1.016** | **1.02** | **1.02** | 0.996 | **1.01** | **1.017** | **1.009** | **1.014** | **1.014** | 0.99 | 1 | 0.99 | 0.98 |
| Leu | UUA | **1.666** | **1.667** | **1.684** | **1.657** | **1.657** | **1.681** | **1.693** | **1.671** | **1.678** | **1.664** | **1.664** | **1.64** | **1.6** | **1.63** | **1.62** |
|  | UUG | **1.314** | **1.305** | **1.296** | **1.295** | **1.295** | **1.296** | **1.307** | **1.293** | **1.314** | **1.289** | **1.289** | **1.30** | **1.28** | **1.3** | **1.3** |
|  | CUU | **1.283** | **1.282** | **1.273** | **1.287** | **1.287** | **1.288** | **1.26** | **1.261** | **1.243** | **1.273** | **1.273** | **1.27** | **1.28** | **1.27** | **1.29** |
|  | CUC | 0.415 | 0.425 | 0.419 | 0.426 | 0.426 | 0.416 | 0.417 | 0.41 | 0.443 | 0.406 | 0.406 | 0.44 | 0.44 | 0.44 | 0.44 |
|  | CUA | 0.845 | 0.841 | 0.846 | 0.853 | 0.853 | 0.84 | 0.843 | 0.867 | 0.839 | 0.867 | 0.867 | 0.85 | 0.89 | 0.85 | 0.85 |
|  | CUG | 0.477 | 0.48 | 0.482 | 0.483 | 0.483 | 0.479 | 0.48 | 0.498 | 0.483 | 0.5 | 0.5 | 0.50 | 0.51 | 0.5 | 0.5 |
| Lys | AAA | **1.448** | **1.455** | **1.454** | **1.447** | **1.447** | **1.457** | **1.452** | **1.443** | **1.453** | **1.438** | **1.439** | **1.41** | **1.42** | **1.41** | **1.42** |
|  | AAG | 0.552 | 0.545 | 0.546 | 0.553 | 0.553 | 0.543 | 0.548 | 0.557 | 0.547 | 0.563 | 0.561 | 0.59 | 0.58 | 0.59 | 0.58 |
| Met | AUG | 1 | 1 | 1 | 1 | 1 | 1 | 1 | 1 | 1 | 1 | 1 | 1 | 1 | 1 | 1 |
| Phe | UUU | **1.267** | **1.26** | **1.265** | **1.266** | **1.266** | **1.264** | **1.27** | **1.259** | **1.251** | **1.264** | **1.264** | **1.2** | **1.19** | **1.2** | **1.19** |
|  | UUC | 0.733 | 0.74 | 0.735 | 0.734 | 0.734 | 0.736 | 0.73 | 0.741 | 0.749 | 0.736 | 0.736 | 0.8 | 0.81 | 0.8 | 0.81 |
| Pro | CCU | **1.631** | **1.64** | **1.64** | **1.645** | **1.645** | **1.64** | **1.64** | **1.578** | **1.572** | **1.584** | **1.584** | **1.58** | **1.55** | **1.58** | **1.58** |
|  | CCC | 0.85 | 0.848 | 0.848 | 0.851 | 0.851 | 0.848 | 0.848 | 0.858 | 0.883 | 0.846 | 0.846 | 0.86 | 0.87 | 0.86 | 0.86 |
|  | CCA | **1.143** | **1.131** | **1.131** | **1.121** | **1.121** | **1.131** | **1.131** | **1.093** | **1.103** | **1.106** | **1.106** | **1.16** | **1.13** | **1.16** | **1.16** |
|  | CCG | 0.376 | 0.382 | 0.382 | 0.383 | 0.383 | 0.382 | 0.382 | 0.471 | 0.441 | 0.464 | 0.464 | 0.4 | 0.45 | 0.4 | 0.4 |
| Ser | UCU | **1.809** | **1.811** | **1.805** | **1.816** | **1.816** | **1.782** | **1.817** | **1.846** | **1.882** | **1.825** | **1.825** | **1.81** | **1.83** | **1.8** | **1.78** |
|  | UCC | 0.955 | 0.951 | 0.98 | 0.986 | 0.986 | 0.978 | 0.977 | 0.932 | 0.9 | 0.944 | 0.944 | **1.06** | **1.04** | **1.07** | **1.07** |
|  | UCA | **1.245** | **1.235** | **1.237** | **1.226** | **1.226** | **1.246** | **1.233** | **1.246** | **1.309** | **1.244** | **1.244** | **1.23** | **1.27** | **1.24** | **1.24** |
|  | UCG | 0.473 | 0.476 | 0.458 | 0.452 | 0.452 | 0.48 | 0.457 | 0.434 | 0.4 | 0.427 | 0.427 | 0.49 | 0.44 | 0.48 | 0.51 |
|  | AGU | **1.264** | **1.262** | **1.264** | **1.263** | **1.263** | **1.265** | **1.251** | **1.274** | **1.273** | **1.289** | **1.289** | **1.16** | **1.17** | **1.15** | **1.16** |
|  | AGC | 0.255 | 0.265 | 0.256 | 0.258 | 0.258 | 0.249 | 0.265 | 0.268 | 0.236 | 0.272 | 0.272 | 0.26 | 0.25 | 0.26 | 0.24 |
| Thr | ACU | **1.486** | **1.5** | **1.469** | **1.49** | **1.49** | **1.469** | **1.453** | **1.497** | **1.519** | **1.528** | **1.528** | **1.42** | **1.47** | **1.42** | **1.42** |
|  | ACC | 0.651 | 0.64 | 0.665 | 0.645 | 0.645 | 0.653 | 0.663 | 0.659 | 0.653 | 0.676 | 0.676 | 0.66 | 0.67 | 0.66 | 0.65 |
|  | ACA | **1.383** | **1.395** | **1.399** | **1.396** | **1.396** | **1.399** | **1.419** | **1.389** | **1.401** | **1.353** | **1.353** | **1.42** | **1.39** | **1.4** | **1.41** |
|  | ACG | 0.48 | 0.465 | 0.466 | 0.469 | 0.469 | 0.478 | 0.465 | 0.455 | 0.427 | 0.443 | 0.443 | 0.51 | 0.46 | 0.52 | 0.52 |
| Trp | UGG | 1 | 1 | 1 | 1 | 1 | 1 | 1 | 1 | 1 | 1 | 1 | 1 | 1 | 1 | 1 |
| Tyr | UAU | **1.629** | **1.627** | **1.621** | **1.616** | **1.616** | **1.636** | **1.615** | **1.587** | **1.57** | **1.596** | **1.596** | **1.62** | **1.65** | **1.61** | **1.63** |
|  | UAC | 0.371 | 0.373 | 0.379 | 0.384 | 0.384 | 0.364 | 0.385 | 0.413 | 0.43 | 0.404 | 0.404 | 0.38 | 0.35 | 0.39 | 0.37 |
| Val | GUU | **1.582** | **1.591** | **1.581** | **1.586** | **1.586** | **1.572** | **1.586** | **1.496** | **1.522** | **1.507** | **1.507** | **1.48** | **1.4** | **1.48** | **1.47** |
|  | GUC | 0.539 | 0.538 | 0.543 | 0.541 | 0.541 | 0.54 | 0.544 | 0.582 | 0.555 | 0.568 | 0.568 | 0.61 | 0.64 | 0.61 | 0.6 |
|  | GUA | **1.284** | **1.275** | **1.251** | **1.261** | **1.261** | **1.29** | **1.254** | **1.258** | **1.286** | **1.264** | **1.264** | **1.26** | **1.29** | **1.26** | **1.3** |
|  | GUG | 0.596 | 0.596 | 0.625 | 0.613 | 0.613 | 0.598 | 0.615 | 0.665 | 0.637 | 0.661 | 0.661 | 0.66 | 0.67 | 0.66 | 0.63 |
| TER | UAA | **1.444** | **1.5** | **1.5** | **1.44** | **1.44** | **1.615** | **1.5** | **1.154** | **1.111** | **1.138** | **1.138** | **1.63** | **1.41** | **1.63** | **1.71** |
|  | UAG | **1** | **1.038** | **1.038** | **1.08** | **1.08** | **1.038** | **1.038** | **1.269** | **1.222** | **1.138** | **1.138** | **1.03** | **1.15** | **1.03** | **1.03** |
|  | UGA | 0.556 | 0.462 | 0.462 | 0.48 | 0.48 | 0.346 | 0.462 | 0.577 | 0.667 | 0.724 | 0.724 | 0.34 | 0.44 | 0.34 | 0.26 |

**Table S6** **Mitochondrial protein-coding genes in *N. listeroides* complex.**

|  | **LJL01** | **LJL02** | **MMD** | **LLZ02** | **HLZ** | **LLZ01** | **LCY01** | **LCY02** | **HHL01** | **HHL02** | **LLJ** | **MLJ** | **LGZ** | **LGS** | **HWQ** |
| --- | --- | --- | --- | --- | --- | --- | --- | --- | --- | --- | --- | --- | --- | --- | --- |
| *atp1* | + | + | + | + | + | + | + | + | + | + | + | + | + | + | + |
| *atp4* | + | + | + | + | + | + | + | + | + | + | + | + | + | + | + |
| *atp6* | + | + | + | + | + | + | + | + | + | + | + | + | + | + | + |
| *atp8* | + | + | + | + | + | + | + | + | + | + | + | + | + | + | + |
| *atp9* | + | + | + | + | + | + | + | + | + | + | + | + | + | + | + |
| *ccmB* | + | + | + | + | + | + | + | + | + | + | + | + | + | + | + |
| *ccmC* | + | + | + | + | + | + | + | + | + | + | + | + | + | + | + |
| *ccmFc* | + | + | + | + | + | + | + | + | + | + | + | + | + | + | + |
| *ccmFn* | + | + | + | + | + | + | + | + | + | + | + | + | + | + | + |
| *cob* | + | + | + | + | + | + | + | + | + | + | + | + | + | + | + |
| *cox1* | + | + | + | + | + | + | + | + | + | + | + | + | + | + | + |
| *cox2* | + | + | + | + | + | + | + | + | + | + | + | + | + | + | + |
| *cox3* | + | + | + | + | + | + | + | + | + | + | + | + | + | + | + |
| *matR* | + | + | + | + | + | + | + | + | + | + | + | + | + | + | + |
| *mttB* | + | + | + | + | + | + | + | + | + | + | + | + | + | + | + |
| *nad1* | + | + | + | + | + | + | + | + | + | + | + | + | + | + | + |
| *nad2* | + | + | + | + | + | + | + | + | + | + | + | + | + | + | + |
| *nad3* | + | + | + | + | + | + | + | + | + | + | + | + | + | + | + |
| *nad4* | + | + | + | + | + | + | + | + | + | + | + | + | + | + | + |
| *nad4L* | + | + | + | + | + | + | + | + | + | + | + | + | + | + | + |
| *nad5* | + | + | + | + | + | + | + | + | + | + | + | + | + | + | + |
| *nad6* | + | + | + | + | + | + | + | + | + | + | + | + | + | + | + |
| *nad7* | + | + | + | + | + | + | + | + | + | + | + | + | + | + | + |
| *nad9* | + | + | + | + | + | + | + | + | + | + | + | + | + | + | + |
| *rpl2* | + | + | + | + | + | + | + | + | + | + | + | + | + | + | + |
| *rpl5* | + | + | + | + | + | + | + | + | + | + | + | + | + | + | + |
| *rpl6* | - | - | - | - | - | - | - | - | - | - | - | - | - | - | - |
| *rpl10* | - | - | - | - | - | - | - | - | - | - | - | - | - | - | - |
| *rpl16* | + | + | + | + | + | + | + | + | + | + | + | + | + | + | + |
| *rps1* | + | + | + | + | + | + | + | + | + | + | + | + | + | + | + |
| *rps2* | + | + | + | + | + | + | + | + | + | + | + | + | + | + | + |
| *rps3* | + | + | + | + | + | + | + | + | + | + | + | + | + | + | + |
| *rps4* | + | + | + | + | + | + | + | + | + | + | + | + | + | + | + |
| *rps7* | + | + | + | + | + | + | + | + | + | + | + | + | + | + | + |
| *rps10* | + | + | + | + | + | + | + | + | + | + | + | + | + | + | + |
| *rps11* | + | + | + | + | + | + | + | + | + | + | + | + | + | + | + |
| *rps12* | + | + | + | + | + | + | + | + | + | + | + | + | + | + | + |
| *rps13* | + | + | + | + | + | + | + | + | + | + | + | + | + | + | + |
| *rps14* | + | + | + | + | + | + | + | + | + | + | + | + | + | + | + |
| *rps19* | + | + | + | + | + | + | + | + | + | + | + | + | + | + | + |
| *sdh3* | - | - | - | - | - | - | - | - | - | - | - | - | - | - | - |
| *sdh4* | - | - | - | - | - | - | - | - | - | - | - | - | - | - | - |
|  |  |  |  |  |  |  |  |  |  |  |  |  |  |  |  |
|  |  |  |  |  |  |  |  |  |  |  |  |  |  |  |  |
|  |  |  |  |  |  |  |  |  |  |  |  |  |  |  |  |
|  |  |  |  |  |  |  |  |  |  |  |  |  |  |  |  |
|  |  |  |  |  |  |  |  |  |  |  |  |  |  |  |  |

**
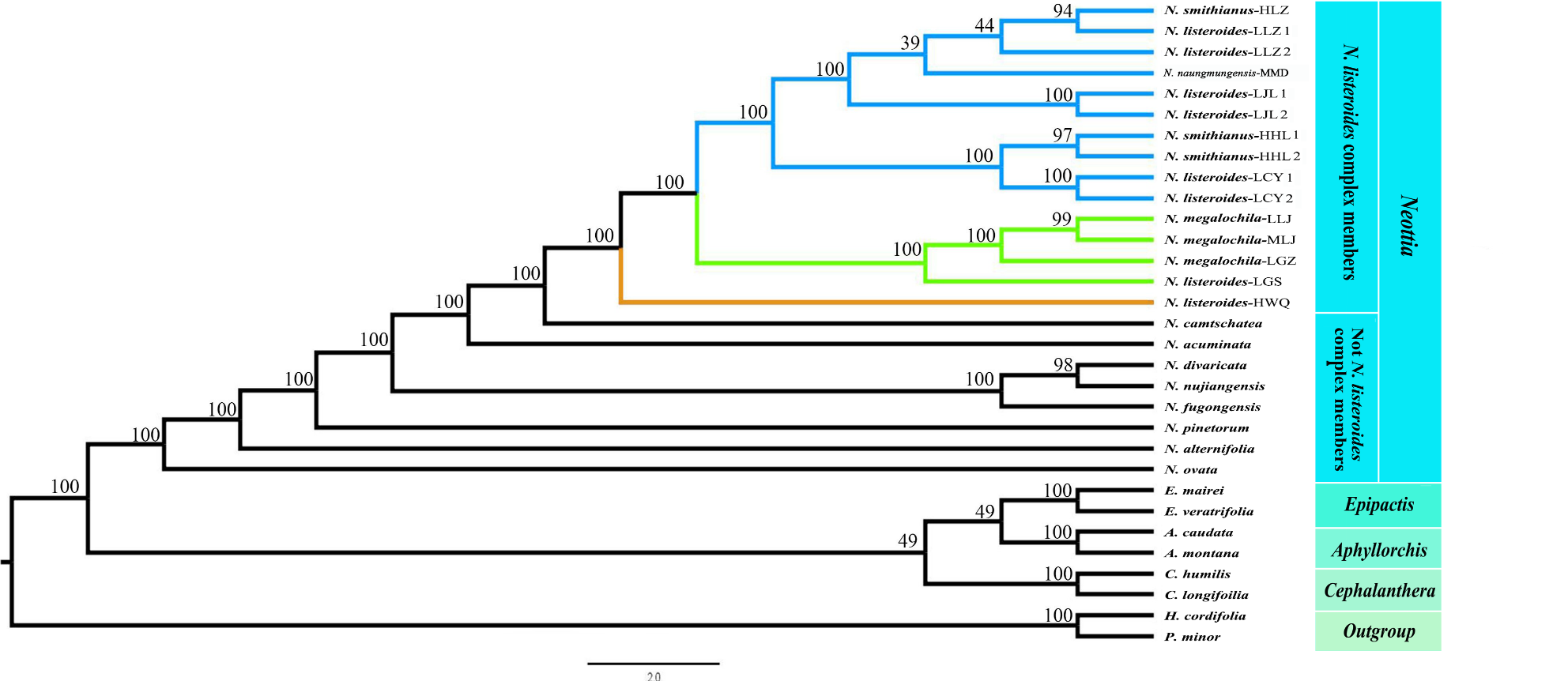
Fig. S1** Phylogenetic relationships based on maximum likelihood (ML) analysis of protein-coding sequences of plastid genome (ptCDS). Numbers above branches represent bootstrap support. *N. listeroides* complex members were divided into three clades: Pine Clade (blue branch) including samples from pine-broadleaf mixed forests; Fir Clade (green branch) including samples from alpine fir forests and For Clade (yellow branch) including sample from alpine fir--broadleaf mixed forest.

**
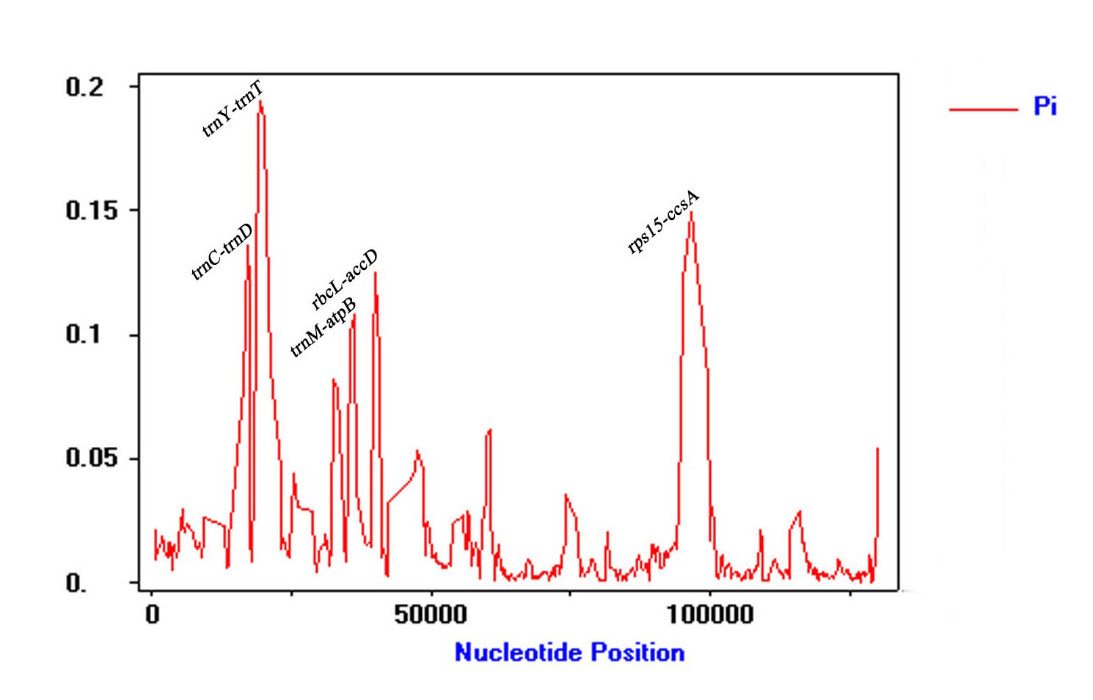
Fig. S2** Mutation hotspots in plastomes of the *N. listreoides* complex.


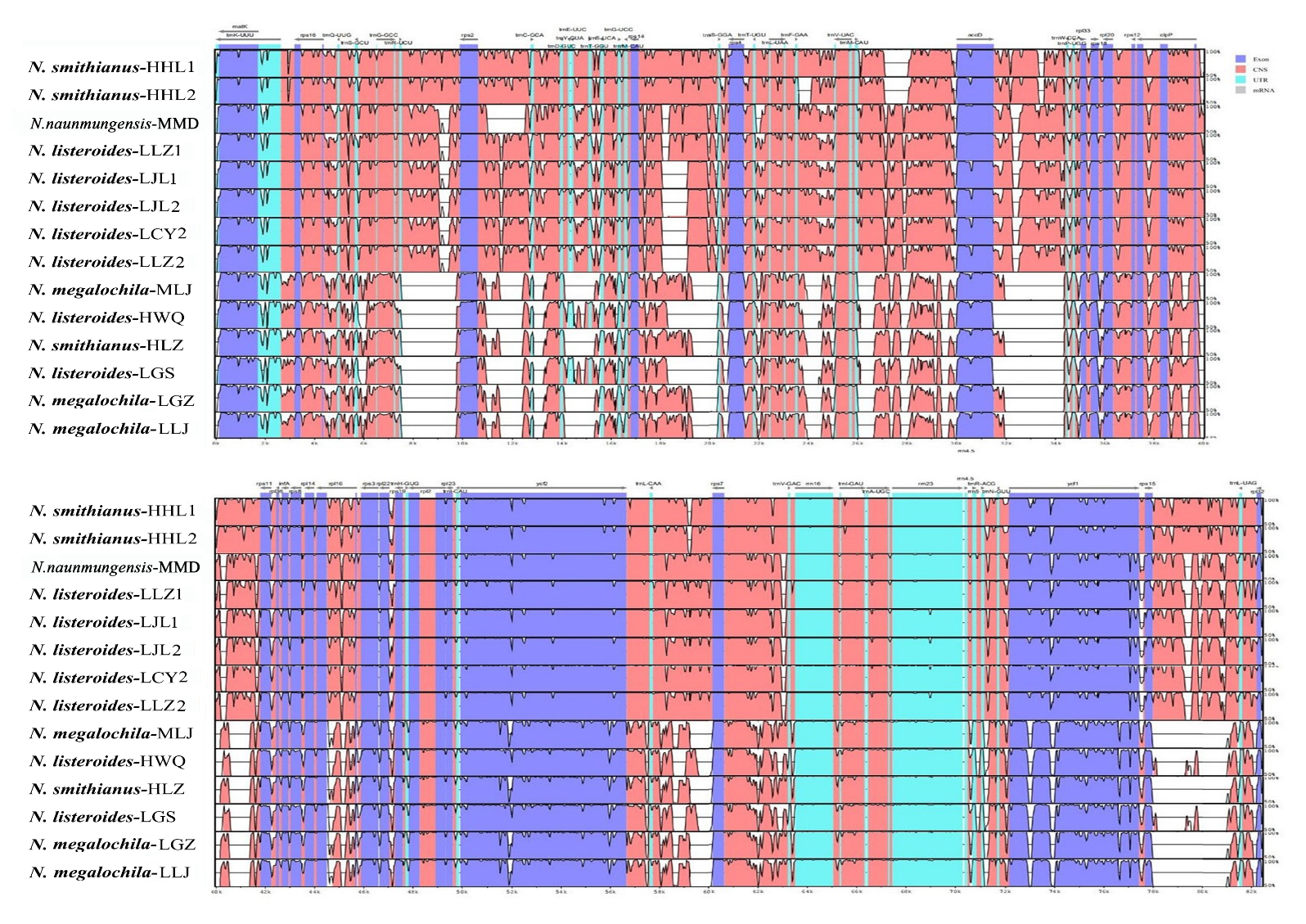
**Fig. S3** Sequence identity of plastomes of *N. listeroides* complex members (LCY-1 as the reference). The vertical scale represents the percentage of identity between 50% and 100%. The horizonal axis indicates the coordinates within the plastomes.

**
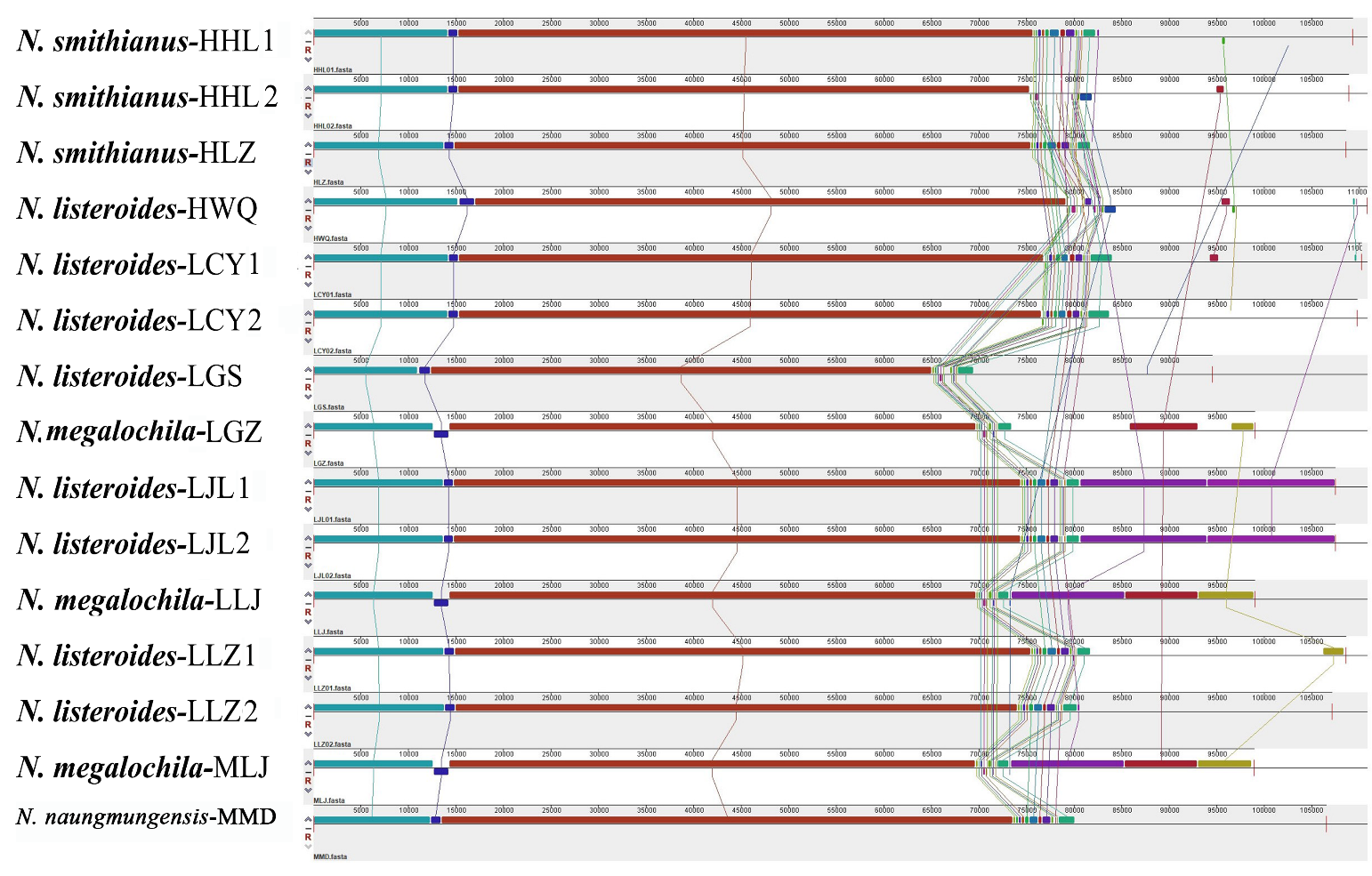
Fig. S4** Colinear analysis of plastomes of the *N. listeroides* complex. Color bands are locally-collinear blocks, representing homologous gene clusters. Within each block, similarity profiles of sequences corresponding to the average conservative level was shown.

**
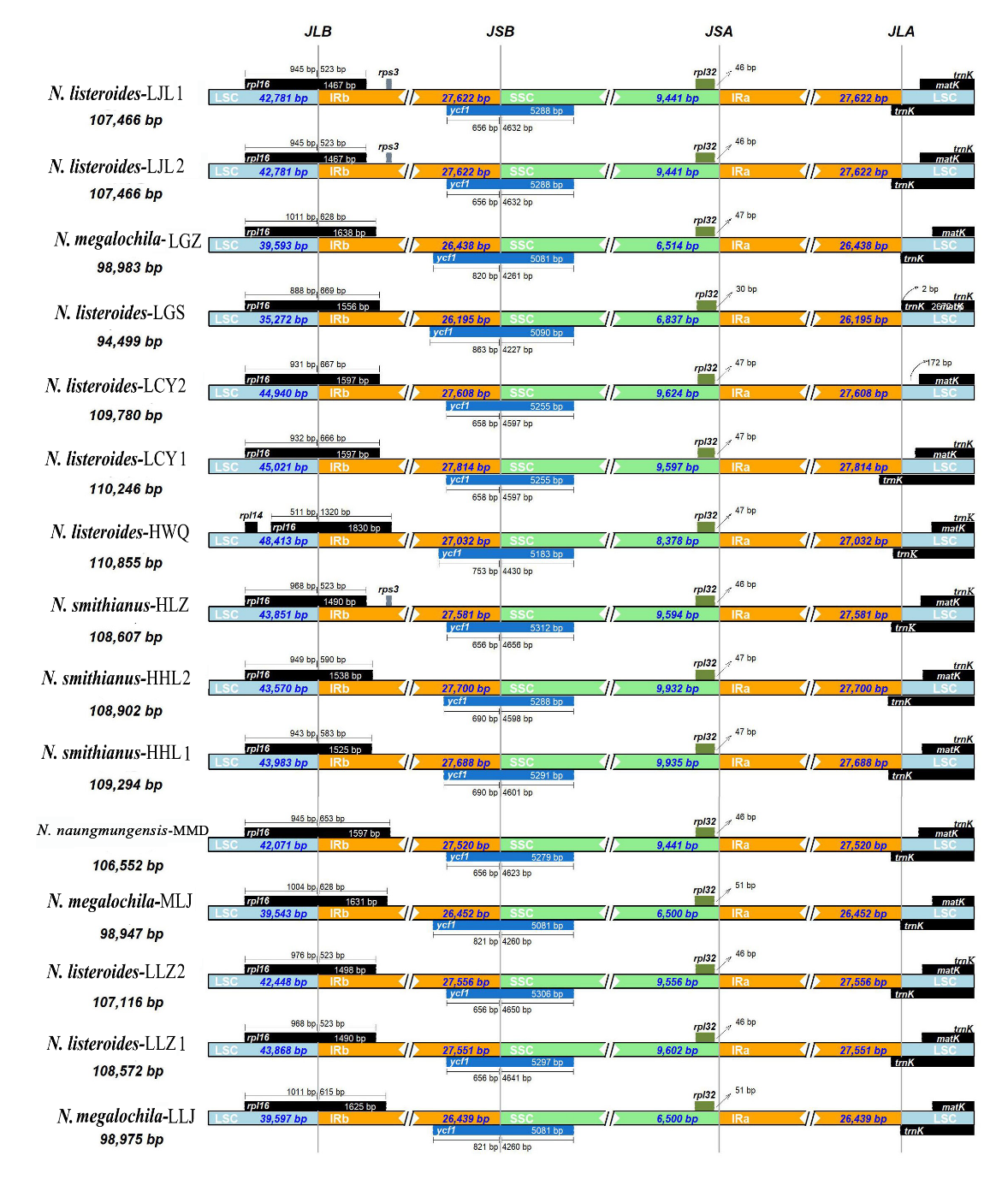
Fig. S5** Comparison of LSC, SSC, and IR border regions among 15 *N. listeroides* complex plastomes. Colored boxes for genes represent the gene position. Gene and region lengths are not to scale.


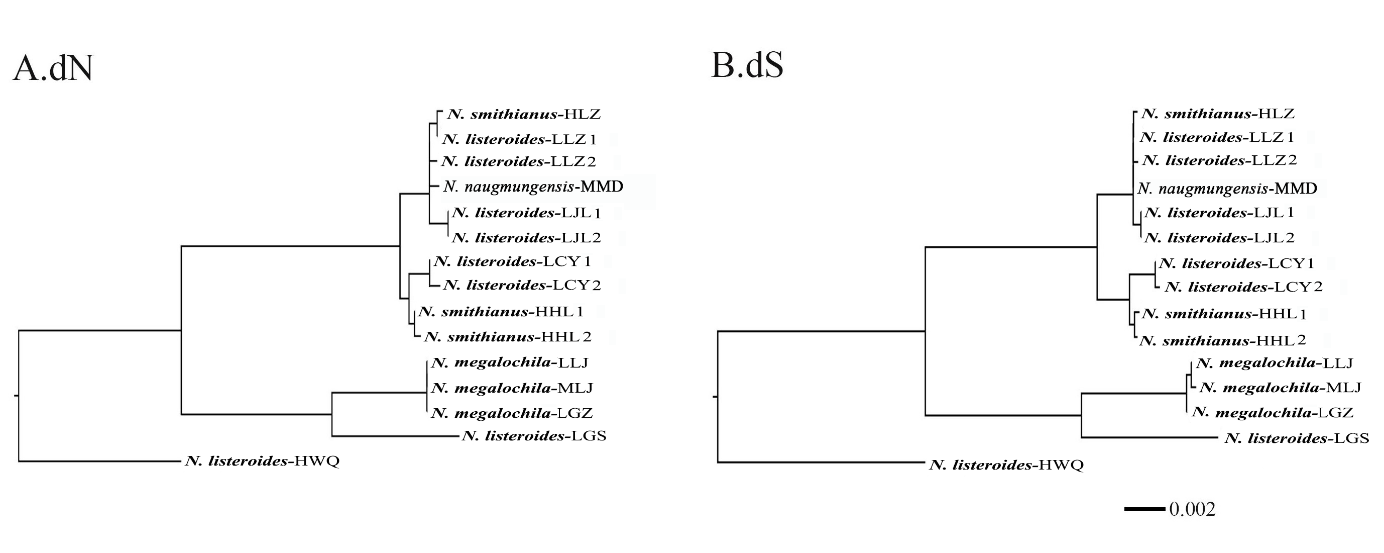


**Fig. S6** Branch length of non-synonymous (dN) and synonymous (dS) substitution rates of *N. listeroides* complex members.
